# M﻿etagenomic insights into the microbial communities of inert and oligotrophic outdoor pier surfaces of a coastal city

**DOI:** 10.1186/s40168-021-01166-y

**Published:** 2021-11-02

**Authors:** Xinzhao Tong, Marcus H. Y. Leung, Zhiyong Shen, Justin Y. Y. Lee, Christopher E. Mason, Patrick K. H. Lee

**Affiliations:** 1 School of Energy and Environment, City University of Hong Kong, Hong Kong SAR, China; 2 Department of Physiology and Biophysics, Weill Cornell Medicine, New York, NY USA; 3 The HRH Prince Alwaleed Bin Talal Bin Abdulaziz Alsaud Institute for Computational Biomedicine, Weill Cornell Medicine, New York, NY USA; 4 The WorldQuant Initiative for Quantitative Prediction, Weill Cornell Medicine, New York, NY USA; 5 The Feil Family Brain and Mind Research Institute, Weill Cornell Medicine, New York, NY USA; 6 State Key Laboratory of Marine Pollution, City University of Hong Kong, Hong Kong SAR, China

**Keywords:** Outdoor surfaces, Piers, Metagenomic sequencing, Metagenome-assembled genomes, Functional traits, Secondary biosynthetic capacity

## Abstract

**Background:**

Studies of the microbiomes on surfaces in built environment have largely focused on indoor spaces, while outdoor spaces have received far less attention. Piers are engineered infrastructures commonly found in coastal areas, and due to their unique locations at the interface between terrestrial and aquatic ecosystems, pier surfaces are likely to harbor interesting microbiology. In this study, the microbiomes on the metal and concrete surfaces at nine piers located along the coastline of Hong Kong were investigated by metagenomic sequencing. The roles played by different physical attributes and environmental factors in shaping the taxonomic composition and functional traits of the pier surface microbiomes were determined. Metagenome-assembled genomes were reconstructed and their putative biosynthetic gene clusters were characterized in detail.

**Results:**

Surface material was found to be the strongest factor in structuring the taxonomic and functional compositions of the pier surface microbiomes. Corrosion-related bacteria were significantly enriched on metal surfaces, consistent with the pitting corrosion observed. The differential enrichment of taxa mediating biodegradation suggests differences between the metal and concrete surfaces in terms of specific xenobiotics being potentially degraded. Genome-centric analysis detected the presence of many novel species, with the majority of them belonging to the phylum Proteobacteria. Genomic characterization showed that the potential metabolic functions and secondary biosynthetic capacity were largely correlated with taxonomy, rather than surface attributes and geography.

**Conclusions:**

Pier surfaces are a rich reservoir of abundant novel bacterial species. Members of the surface microbial communities use different mechanisms to counter the stresses under oligotrophic conditions. A better understanding of the outdoor surface microbiomes located in different environments should enhance the ability to maintain outdoor surfaces of infrastructures.

Video Abstract

**Supplementary Information:**

The online version contains supplementary material available at 10.1186/s40168-021-01166-y.

## Introduction

Microorganisms are ubiquitous in both the natural and engineered environments. Due to strong dispersal and adaptive capacities, some microbial taxa are widely distributed in diverse ecosystems [1]. Recent studies [2, 3] have shown that the indoor and outdoor surfaces of the built environment are reservoirs of microbial assemblages from multiple sources such as humans and nearby surroundings. The microbial communities on indoor surfaces are influenced by geographical location [4], building function [5], building design [6], cleaning practices [7], human occupancy [8], and occupant activities [9]. Indoor surfaces not only passively receive microbes, but also facilitate microbial growth when moisture is available [10]. In an occupied indoor space, different surface types harbor distinct microbial communities [11], which is largely due to contact by occupants and the subsequent transfer of microbes [12]. The interactions between microbes and surfaces are affected by many factors such as surface hydrophobicity, charge, topography, and other physicochemical attributes [13–16]. Consequently, the abundances of specific taxa differ depending on the type of surfaces and materials [17, 18]. Similarly, the metabolic functions of surface microbial communities and the synthesized metabolites also vary by surface type [11, 17] and materials [10].

Unlike indoor surfaces, outdoor surfaces are often exposed to uncontrolled and harsh environmental conditions, such as intense ultraviolet light, fluctuating temperature, desiccation, and poor nutrient supply. These conditions and stressors not only induce esthetic deterioration of the surfaces (e.g., corrosion), but also threaten the survival of microbial residents [19]. However, some of the microbes can adapt to and survive such stresses by a variety of strategies [20]. For example, the transcription factors OxyR and SoxRS in bacterial cells can be activated in response to oxidative stress [21], and the high-osmolarity glycerol mitogen-activated protein kinase signaling pathway in yeasts can also be induced for adaptation to osmotic changes [22]. Therefore, some microbial residents of surfaces are considered stress-tolerant and they may in turn participate in biochemical processes that influence various properties of the surfaces [23]. For example, on inert surfaces such as stone and steel, some microbes can induce or accelerate corrosion [24], while other microbes can prevent biodeterioration [25]. The taxonomic composition of microbes residing on corroded steel surfaces has been found to vary by surface type, surface material, and environmental conditions (e.g., salinity), but the composition of their metabolic functions is relatively conserved [26].

Piers are engineered built environments commonly found in coastal areas, providing access to offshore areas. Located outdoors at the interface between terrestrial and aquatic ecosystems, pier surfaces are colonized by microorganisms that are passively deposited from both environments, making the surfaces a unique platform for microbial exchanges. Similar to indoor surfaces, humans may transfer their microbial assemblages onto pier surfaces via contact while using the infrastructures [27]. However, unlike indoor surfaces, pier surfaces are exposed to external stressors from the natural elements, especially seawater, which contain various ions (e.g., chloride and sulfate) that can cause corrosion [28]. The open and outdoor nature of a pier also makes its surfaces an ideal sink for the deposition of marine and atmospheric pollutants [29, 30]. Collectively, pier surfaces are unique habitats for microbial populations from natural and anthropogenic sources, which are under constant stresses from the surroundings.

Despite the widespread use of piers, the microbiomes on pier surfaces have not been extensively investigated. Specifically, the taxonomic and functional compositions of the pier microbiomes, the mechanisms they use to withstand stresses, and the influences of different physical attributes (e.g., surface material and type) and environmental factors (e.g., temperature and relative humidity) are poorly understood. In this study, the metagenomes of samples collected from four types of concrete or metal surfaces from nine piers along the coastline of Hong Kong were analyzed. The determinants governing the taxonomic compositions and metabolic functions and the microbial sources that contributed to the pier surface microbiomes were identified. Metagenome-assembled genomes (MAGs) were reconstructed from the surface microbiomes, including many belonging to novel species, and their putative biosynthetic gene clusters (BGCs) were characterized. This study shows that outdoor pier surfaces are a rich reservoir of unexplored microbial genomes and metabolic functions, and the insights gained from this study could aid maintenance of engineered infrastructures.

## Results

### Taxonomic overview of pier surface microbiomes

**Fig. 1:**
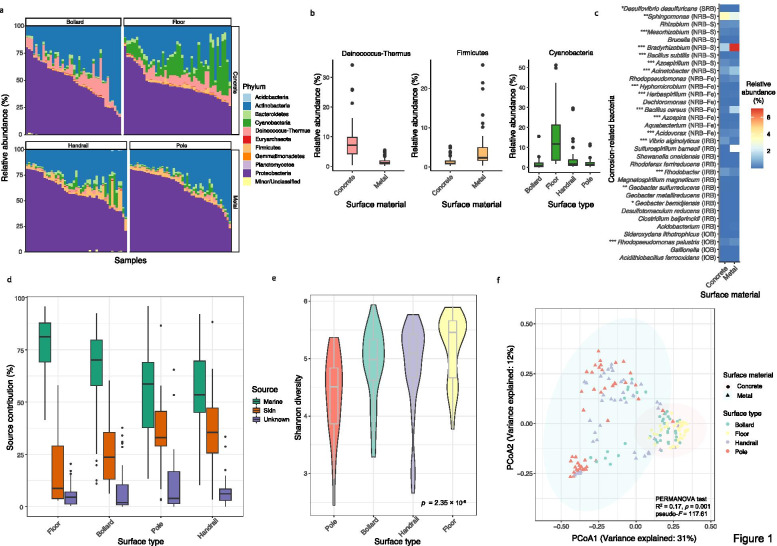
Composition and diversity of pier surface microbiomes. **a** Top 10 phyla across the four surface types. Other phyla were grouped into “Minor/Unclassified.” **b** The three phyla that were differentially enriched between different surface types and materials. **c** The mean relative abundances of the corrosion-related bacteria identified on the metal and concrete surfaces. The full names of the microbial corrosion mechanism abbreviations are indicated in the “Materials and methods” section. The Mann–Whitney test was applied to determine the differential enrichment of corrosion-related bacteria between concrete and metal surfaces (***p < 0.001, **0.001 < *p* < 0.01, *0.01 < *p* < 0.05). **d** Contributions by local marine and human skin sources to pier surface microbiomes. **e** Shannon diversity of microbiomes across different surface types. **f** Principal coordinate analysis of surface microbiomes based on the species-level abundance matrix ordinated by the Bray–Curtis dissimilarity metric. The normal confidence ellipses indicate the confidence level at 95%

Of the 175 outdoor pier surface samples analyzed, 99.5% of the reads on average were annotated as bacteria, 0.34% as viruses, and 0.17% as archaea. At the phylum level, the pier surface microbiomes were dominated by Proteobacteria and Actinobacteria (Fig. 1a). Specifically, Deinococcus–Thermus was significantly enriched on concrete surfaces (Mann–Whitney [MW] test, *p* = 1.43 × 10^− 19^), while metal surfaces were dominated by Firmicutes (MW test, *p* = 1.48 × 10^− 15^). In addition, the marine cyanobacterial populations were significantly more abundant on the floor than on other surface types (Kruskal–Wallis [KW] post hoc test, *p* < 0.05 for all comparisons) (Fig. 1b). At the species level, 17 bacterial species were significantly associated with surface materials (Additional file 1: Fig. S1). For metal surfaces, species that are human- and environment-associated, especially methylotrophic bacteria, were significantly enriched. Meanwhile, stone-dwelling and photosynthetic bacteria were significantly enriched on concrete surfaces.

Of the 56 representative corrosion-related bacteria on the compiled list [31–34] (Additional file 2: Table S1), 33 could be identified in at least one of the pier samples. Taxa from each of the five mechanistic groups delineated by corrosion-causing mechanisms were significantly enriched on metal and concrete surfaces (Fig. 1c). For example, *Bacillus cereus* (MW test, *p* = 2.29 × 10^− 12^), which is in the group of nitrate-reducing bacteria associated with the redox cycling of iron (NRB-Fe), and species belonging to *Acinetobacter* (*p* = 1.69 × 10^− 8^) and *Bradyrhizobium* (4.05 × 10^− 17^), which are in the group of siderophore-producing NRB (NRB-S), were significantly enriched on metal surfaces. However, members of *Sphingomonas* (NRB-S) were significantly enriched on concrete (*p* = 0.01).

With the observation that marine- and human-associated microbial taxa were present on pier surfaces, source tracking was performed to query the extent to which marine and human microbial sources contributed to the pier surface microbiomes. The source tracking results showed that the marine environment (average 62.9 ± 20.3%) and human skin (average 29.4 ± 18.0%) were the major microbial sources of all of the pier surface microbiomes. The floor harbored more species derived from marine sources than did other types of surfaces, while skin sources were expectedly more abundant on poles and handrails (Fig. 1d). This result is consistent with indoor microbial studies, where the floor is generally dominated by environmental species [35], while surfaces frequently touched by humans are dominated by skin commensals [9]. The contribution of unknown sources was low across all surface types (7.7 ± 9.9%).

### Surface attributes structured the diversity and composition of pier surface microbiomes

To identify parameters that shaped the within-sample diversity of pier surface microbiomes, a stepwise Akaike information criterion (AIC) model selection scheme based on the Shannon diversity index was performed. The optimal model identified two significant parameters and one interactive parameter, which together explained 69% of the within-sample diversity variance (Additional file 3: Table S2). Surface type (pseudo-*F* = 20.27, *p* = 1.0 × 10^− 10^, *R*^2^ = 0.16) and sampling location (pseudo-*F* = 3.43, *p* = 0.001, *R*^2^ = 0.07) were predicted to be the two most influential parameters. Specifically, the floor microbiomes displayed the highest diversity among the four surface types, while the pole microbiomes had the lowest (KW test, *p* = 2.35 × 10^− 6^, Fig. 1e).

Furthermore, to identify the most important parameters driving the compositional differences between surface microbiomes, a PERMANOVA test was applied to a stepwise AIC model selection scheme. The optimal model identified three independent parameters and one interactive parameter, which together explained 80% of the between-sample variance (Additional file 4: Table S3). Surface material (pseudo-*F* = 117.61, *R*^2^ = 0.17, *p* = 0.001) was the most important parameter driving the compositional differences between surface microbiomes (Fig. 1f), followed by surface type (pseudo-*F* = 21.55, *R*^2^ = 0.06, *p* = 0.001) and sampling location (pseudo-*F* = 18.21, *R*^2^ = 0.21, *p* = 0.001). Overall, these results highlighted the importance of surface attributes in structuring the diversity and composition of pier surface microbiomes.

### Correlations between taxonomy and functions of pier surface microbiomes

The functional composition of surface microbiomes was found to be significantly correlated with the species-level taxonomic composition (Procrustes test, *p* = 0.001, correlation 0.7464), suggesting that samples with a similar taxonomic composition tended to have a similar functional composition. Because material was the strongest factor in structuring the composition of surface microbiomes, the species-level contributions to the functional shifts of surface microbiomes were further quantified for the respective concrete and metal surface microbiomes. Twenty-eight and 69 metabolic pathways were found to be significantly enriched on metal and concrete surfaces, respectively, with the majority of them encoding housekeeping functions. Interestingly, pathways related to energy metabolism and xenobiotics biodegradation and metabolism were also enriched.

Notably, for metal surfaces, *Micrococcus luteus*, *Bacillus cereus*, and a few *Bradyrhizobium* species were the major drivers of enrichment of xenobiotics biodegradation pathways (Additional file 5: Fig. S2). For concrete surfaces, the enrichment of xenobiotics biodegradation pathways was driven predominantly by species including *Deinococcus* sp. Strain NW-56 and three stone-dwelling Actinobacteria including *Blastococcus saxobsidens*, *Modestobacter marinus*, and *Geodermatophilus obscurus* [36]. In addition, diverse species on concrete surfaces were associated with energy metabolism pathways, with a few cyanobacterial species involved in photosynthetic carbon fixation pathways, consistent with their photosynthetic physiology [37].

### Surface material determined the functional variations of pier surface microbiomes

To understand how the microbiomes responded to the oligotrophic conditions of pier surfaces, the functional profiles of contigs in each sample were characterized following read assembly. Functional gene annotation based on the cluster of orthologous group (COG) categories revealed differential enrichment of genes in metagenomes from different surface materials (Additional file 6: Fig. S3). Significant differences were detected between metal and concrete surface metagenomes for the majority of COG categories, with the exception of carbohydrate transport and metabolism [G], lipid transport and metabolism [I], and signal transduction mechanisms [T] (MW test, *p* > 0.05 for these three cases). No significant differences for any of the COG categories were detected between microbiomes residing on the two types of metal surfaces, while significant differences between microbiomes on the two types of concrete surfaces were only found for four COG categories [C, E, I, M]. These results suggest that surface material is more important than the surface type in driving microbial functions.

**Fig. 2:**
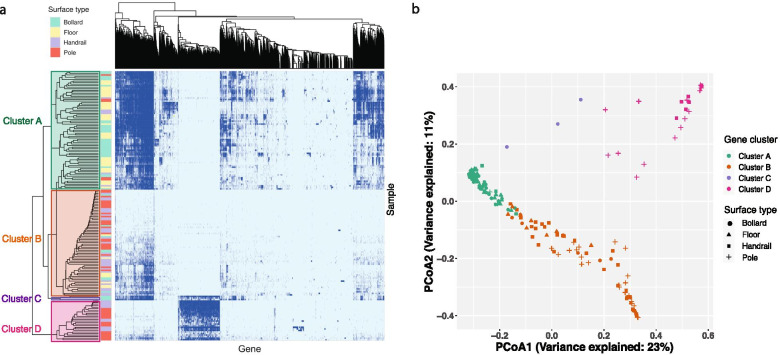
Surface materials governed the functional traits of pier surface microbiomes. **a** Two-way hierarchical clustering of all genes identified in the contigs of each sample. Genes that are present or absent are indicated by dark and light blue colors, respectively. Genes (column) were hierarchically clustered based on their presence/absence in the samples. The four gene clusters of the vertical dendrogram are highlighted. **b** Principal coordinate analysis of the binary Jaccard distance based on the presence/absence of genes in the surface microbiomes. Each point represents a sample

Furthermore, we hypothesized that microbiomes from the same material possessed similar gene repertoires. To test this hypothesis, a two-way hierarchical clustering analysis was performed on the Jaccard distance index between the samples regardless of surface material and type (Fig. 2a). The clustering resulted in four distinct gene clusters (Fig. 2b). Cluster A was dominated by genes sourced from concrete surfaces (66 out of 77), cluster B mostly contained genes from metal surfaces (63 out of 69), cluster C contained genes derived from three bollard samples at a single location, and cluster D comprised genes from 26 metal samples from six locations. The hierarchical clustering results were further supported by the supervised random forest classifier, which yielded an overall out-of-bag error score of 3.4%. These results suggest that surface material regulated the functional traits of surface microbiomes, with microbes from the same material possessing similar metabolic functions.

The clear separation of cluster D from the other clusters suggested the presence of a unique gene repertoire (Fig. 2b). A detailed analysis of cluster D revealed 1263 genes (with a prevalence of > 75% in the samples in this cluster and < 5% in the samples of other clusters), the majority of which belonged to the COG categories [O] (post-translational modification, protein turnover, and chaperones), [U] (intracellular trafficking, secretion, and vesicular transport), [T] (signal transduction mechanisms), and [J] (translation, ribosomal structure, and biogenesis) (Additional file 7: Fig. S4). Previous studies have shown that bacterial genomes containing specific functional gene inventories enable their survival in particular ecological niches [38]. Therefore, the group of genes in cluster D may confer beneficial adaptive functions on the microbes residing on those particular metal surfaces.

Because the biodegradation pathways for a few xenobiotics were differentially enriched between concrete and metal surfaces (Additional file 5: Fig. S2), we further queried whether KEGG Orthology (KO) identifiers associated with xenobiotics biodegradation and metabolism pathways differed between surface materials. Two hundred and fifty-one KOs encoding xenobiotics metabolism were identified in the pier metagenomes, with significantly more KOs identified on concrete than on metal surfaces (average 86 vs. 30, MW test, *p* = 4.2 × 10^− 17^). In addition, KOs encoding xenobiotic metabolism tended to be compositionally more similar in microbiomes from the same than from different surface materials (Additional file 8: Fig. S5). Overall, these results further highlight the role of surface material in determining microbial functions.

Trace metals such as iron are crucial for the survival of microorganisms [39]. As the metal surfaces sampled contained iron, and given the result that the abundance of genes involved in inorganic ion transport and mechanism [P] differed significantly between the different surface materials and types (Additional file 6: Fig. S3), we investigated whether such differences could be reflected in genes related to iron metabolism. Metal surfaces contained a higher relative abundance of genes involved in iron acquisition (MW test, *p* = 0.03), while genes involved in iron regulation and storage were significantly more abundant on concrete surfaces (*p* = 0.004 and 1.66 × 10^− 6^, respectively) (Additional file 9: Fig. S6a). Differences in the composition of iron-related protein families between the concrete and metal surface metagenomes were also found (pseudo-*F* = 39.71, *R*^2^ = 0.12, *p* = 0.001, Additional file 9: Fig. S6b). The iron metabolism analysis further reinforced the notion that differences in microbial functions are based on surface material. However, a relatively low abundance of iron-related genes was found in all of the surface metagenomes, with 15 samples even completely lacking these genes, suggesting that iron metabolism is not a major function in the communities, even on metal surfaces. The low relative abundance of genes encoding iron oxidation on both materials is consistent with the low relative abundance of iron-oxidizing bacteria (IOB) (Fig. 1c).

### Novel MAGs were present on pier surfaces and BGCs were associated with taxonomy

**Fig. 3:**
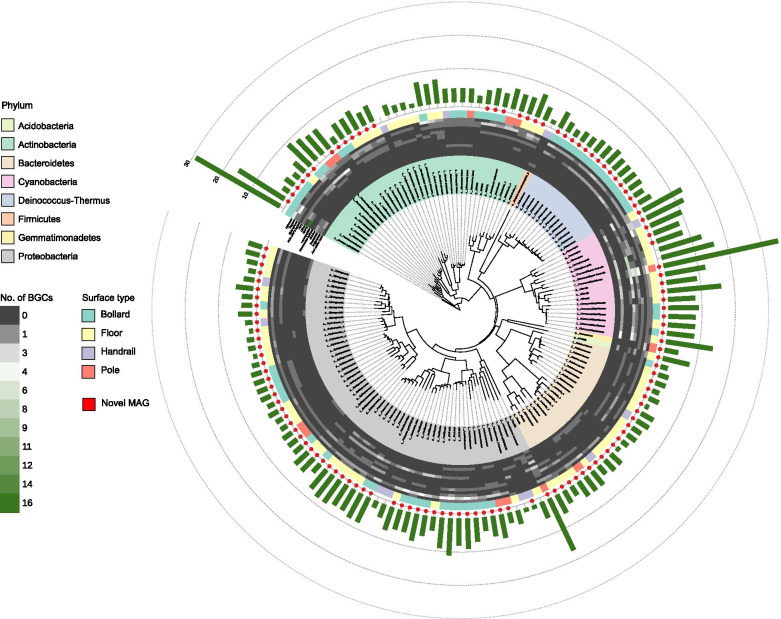
Phylogenetic tree of the 150 MAGs and the putative BGCs found in each MAG. The innermost ring shows the lowest assigned taxonomic rank of the MAGs. The prefix “s” indicates a known species and the prefixes “g” and “f” indicate the lowest possible assigned taxonomic rank at the genus and family levels, respectively. The MAGs that could not be assigned to a known species are indicated by a red dot. The heatmap shows the number of putative BGCs of each of the top 12 known types detected in each MAG. All known types of putative BGCs that were present in < 1% of all of the BGCs were grouped into the “Other” category. The total number of putative BGCs in each MAG is indicated by the green bars in the outer ring

Pier surfaces are considered largely unexplored habitats, so we investigated whether novel genomes could be reconstructed from the microbiomes, and whether the genetic and biosynthetic potential of phylogenetically closely related genomes varied according to surface attributes. One hundred and fifty MAGs (with contamination of ≤5% and completeness of ≥75%) could be reconstructed from the samples. Among them, 67 MAGs were considered as high quality (contamination of ≤5% and completeness of ≥90%) based on the standard established by the Genomic Standards Consortium [40]. Consistent with the taxonomic profiling of the short reads, the reconstructed MAGs predominantly belonged to the phyla Proteobacteria (58/150) and Actinobacteria (37/150) (Fig. 3). By using an average nucleotide identity (ANI) threshold of > 95% for species delineation [41], only 25 of the 150 MAGs could be assigned to a known species and the rest could only be classified to a known genus (99/150) or family (26/150) (Additional file 10: Fig. S7), suggesting the presence of potentially novel species on pier surfaces. Many of the MAGs that could not be classified to the species level were retrieved from concrete surfaces (60/125 from the floor and 59/125 from the bollard) and they belonged to the phyla Proteobacteria (53/125), Actinobacteria (21/125), and Bacteroidetes (21/125).

Functional differences of all of the MAGs between either surface type or geographical location were significant in only half of the COG categories (Additional file 11: Table S4). In contrast, significant differences between phyla were detected in all COG categories except for [Z] (KW test, *p* < 0.05 for all significant comparisons) (Additional file 12: Fig. S8), highlighting the importance of taxonomy in regulating the functional traits of genomes. The MAGs in Actinobacteria contained the highest abundance of genes involved in transcription [K], while the MAGs in Bacteroidetes were abundant in genes encoding cell wall/membrane/envelope biogenesis [M]. The MAGs in Cyanobacteria contained the highest abundance of genes related to signal transduction mechanisms [T] but also the lowest abundance of genes related to metabolic functions such as amino acid transport and metabolism [E] compared with other phyla.

**Fig. 4:**
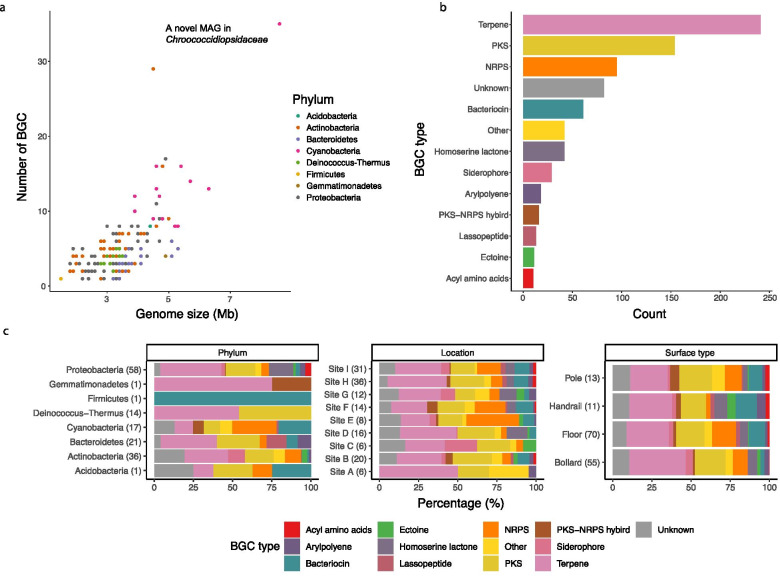
Secondary biosynthetic capacity of the MAGs. **a** Correlation between the genome size and the number of putative BGCs in each MAG. Each data point represents an MAG, colored by phylum classification. **b** Total number of each type of putative BGCs across all MAGs. **c** Relative abundance of the top 12 known BGC types across phylum (left), location (middle), and surface type (right). The total number of putative BGCs in each category is indicated in the brackets. All known types of putative BGCs that were present in < 1% of all of the putative BGCs were grouped into the “Other” category

To further investigate the metabolic capacity between MAGs in different taxonomic groups, the presence of putative BGCs was analyzed. The genome size of the MAGs was found to be linearly correlated with the number of putative BGCs, with the highest number (35 of them) detected in an MAG that could only be annotated to the family *Chroococcidiopsidaceae* (Fig. 4a). The most common type of putative BGC identified among the MAGs was for the synthesis of terpene (Fig. 4b), which is expected given that genes encoding terpene synthases are widely distributed in bacteria [42]. The type and relative abundance of putative BGCs remained relatively similar across surface types and sampling locations but varied significantly across phyla (Fig. 4c and Additional file 13: Table S5). For example, the 17 MAGs belonging to Cyanobacteria, which generally have a larger genome size, harbored significantly more putative BGCs (12 on average) than other phyla (five on average) (post hoc KW test for all comparisons, *p* < 0.05), with the functions of synthesizing bacteriocin and non-ribosomal peptides (NRPs) particularly enriched (Fig. 3). Meanwhile, the putative BGCs encoding acyl-amino acids and homoserine lactones were only detected in 10 and 23 of the MAGs in Proteobacteria, respectively (Fig. 3). Together, these results highlighted the variations in the repertoire of secondary metabolic potentials between genomes from different phyla.

## Discussion

The microbiomes of outdoor surfaces are largely unexplored compared with those of indoor surfaces [9, 11, 43, 44]. As an open system at the interface between marine and terrestrial ecosystems, coastal surfaces are a unique habitat that harbors interesting microbiology. In this study, we characterized the taxonomic profile and functional traits of coastal pier surface microbiomes at both the community and genome levels. The results have shed light on the microbes that are present, the metabolic functions that may facilitate adaptation of these members to the harsh environmental conditions, and the parameters that are associated with taxonomic and functional variations.

### Surface materials drive taxonomic variations and functional shifts in pier microbiomes

Although the surfaces studied here are inert and oligotrophic, surfaces made of different materials inherently vary in their micro-environmental characteristics, such as pH, structure (e.g., cracks for protection against predation), and nutritional availability [45]. From an ecological perspective, the differences between concrete and metal surfaces may impose different stresses on the microbial colonists, resulting in different microbes colonizing different surfaces depending on their ability to adapt to the surface materials. Therefore, the taxonomic composition of the pier surface microbiomes is largely governed by material, and microbial communities that are functionally more similar tend to originate from the same surface material. In fact, the taxonomic and functional compositions of surface microbiomes are strongly congruent, suggesting that variations in the surface microbial composition give rise to shifts in metabolic functions of the microbiome. A similar correlation between the taxonomic composition and the associated resistance potentials and metabolomes has also been reported in other microbial communities of diverse ecosystems [46–48].

### Enrichment of taxa across surface types and materials

The compositional differences between metal and concrete surface microbiomes imply the differential enrichment of taxa between the two materials. In this study, enrichment of microbial taxa across surface type and material was observed. Members of Cyanobacteria were particularly more abundant on the floor than on other surface types, which is consistent with the floor being most susceptible to be imprinted by aquatic taxa from seawater spray. In addition, Cyanobacteria species are known to be prevalent in aerosols above marine water bodies [49] and the relatively large size of Cyanobacteria cells enhances their deposition on the floor, which is an ideal sink for airborne microbes [50]. Members of Deinococcus-Thermus were significantly more abundant on concrete surfaces than on metal surfaces, possibly because the quartz minerals in concrete promote their attachment to the surface [51]. Three stone-dwelling Actinobacteria species [36] were also significantly enriched on the concrete surfaces, possibly because of the similar surface physicochemical properties between concrete and stone. Members of the class Bacilli in the Firmicutes phylum were particularly enriched on the metal surfaces, thus possibly explaining the observed corrosion on these surfaces. Previous works have found that certain species of the genus *Bacillus*, such as the *B. cereus* found on some of the metal pier surfaces, can accelerate the pitting corrosion of steel surfaces in soils [52, 53] and aquatic environments [24]. Although the corrosion-related bacteria remained at low relative abundances on the pier surfaces, they nevertheless can serve as an indicator that maintenance work is required to preserve the esthetic appearance of the metal surfaces. In addition to the environmental taxa, skin-associated bacteria were found to be enriched on the metal handrails and poles, consistent with human users imprinting their skin microbial signatures onto such surfaces during contact [9, 11, 44].

### Microbial sources that shape the pier surface microbiomes

Consistent with the presence of marine- and human-associated microorganisms on the pier surfaces, source tracking prediction supported the prominent role played by marine and human sources in structuring the surface microbiomes in the open outdoor environment. Nevertheless, other sources that are not considered in this study, due to the lack of available local metagenomics data, could also play a role. Although marine sediment microbiomes were used as a proxy for marine sources, the taxonomic compositions of marine sediment and marine surface water are different [54], with the latter being a major source of surface microbial communities of docked boats [55]. In addition, on pier surfaces, we observed the dominance of members of the class α-Proteobacteria, which may originate from marine environments [56] and also the nearby plants and soils [57]. Therefore, microbes from terrestrial environments could be seeding coastal surfaces through dust deposition following aerosolization [58]. Although a diverse population of microbes are present in ambient air, a recent short-term temporal study revealed that airborne bacteria only contributed minimally to the external surfaces of boats docked in the port [59], suggesting that airborne sources may not be a major source of microbiomes in the open pier environment.

### Differentially abundant taxa contributed to the enrichment of functions between surface materials

Our results show that the taxa enriched on the pier surfaces can result in the enrichment of certain metabolic functions. Interestingly, on the floor, abundant Cyanobacteria contributed to the enrichment of photosynthetic carbon metabolism, which through carbon fixation may provide resources for other microbial members of the community [60], enabling community-wide response in an oligotrophic environment [61]. The differential enrichment of taxa mediating biodegradation between concrete and metal surfaces suggests differences in the specific xenobiotics that are potentially degraded. For example, the *Bradyrhizobium* species that were significantly enriched on metal surfaces could be degrading xenobiotics such as chlorocyclohexane and chlorobenzene [62]. However, the biodegradation of polycyclic aromatic hydrocarbons on concrete surfaces could be mediated by the abundant heterotrophic members of Deinococcus-Thermus [63]. The metabolic potential to degrade the pollutants deposited on the surfaces by specific taxa could create a more suitable environment for members of communities that otherwise may be inhibited by exogenous chemicals.

### Genetic functional traits of MAGs are largely regulated by taxonomy

Genome-wide functional annotation of the reconstructed MAGs revealed that taxonomy played a more important role than surface material and type and geography in structuring the genetic functional traits, with significant differences between genomes across phyla. For example, the genomes of Cyanobacteria are enriched with genes for signal transduction. Bacteria in nutrient-poor environments have been shown to preferentially synthesize proteins essential for their survival [64], a process regulated by a signal-dependent mechanism to ensure genes and proteins are expressed or activated only when needed [65]. Bacteria that are enriched with regulatory genes such as those involved in signal transduction may be better at controlling their metabolic functions, allowing the optimization of growth even under stresses [66]. Therefore, it is reasonable that diverse cyanobacterial taxa were found on the pier surfaces. Members of other phyla such as Actinobacteria are enriched in genes encoding functions related to transcription. Bacterial adaptation to specific niches requires transcriptional re-shaping [67] and the expression of many genes under stresses depends on the transcription processes [68]. Moreover, members of the Gram-negative bacterial phylum Bacteroidetes are enriched in genes for cell wall biogenesis, which is functionally essential for maintaining the integrity of the cellular envelope under stresses [69]. Collectively, these results further suggest that members of the various bacterial phyla found on the pier surfaces tend to possess metabolic strategies to adapt and respond to environmental stresses.

### Biosynthetic capacity of MAGs differs by taxonomy rather than by surface type and geography

In addition to cellular metabolic functions, taxonomy was also important in regulating secondary biosynthetic capacity, in which the type and number of putative BGCs varied significantly across phyla but remained relatively similar across geography and surface material and type. This result is consistent with the recent findings from regional and global soil systems [70, 71], highlighting inherent differences in secondary metabolite biosynthetic potential between bacteria of different phyla in response to similar environmental signals. In the surface microbial communities, members of Cyanobacteria tend to possess a strong capacity to produce bacteriocin and NRPs, both of which possess antimicrobial properties [72, 73]. Although some putative BGCs might be silent in practice and require specific conditions before they are expressed [72], the synthesized small molecules may nonetheless be beneficial to the survival of Cyanobacteria by mediating interactions between surface colonizers. Compared with other phyla, Cyanobacteria has a higher number of putative BGCs, including those of unknown classes, which is consistent with the fact that members of this phylum are a prolific source of natural products and their true metabolic potential is far beyond our current knowledge [74]. The novel cyanobacterial MAG belonging to *Chroococcidiopsidaceae*, which has the highest number of putative BGCs, including five of unknown classes, further reinforces the notion that many novel biochemicals await characterization.

### Limitations and future works

While we have shed light on the coastal surface microbiome in this study, there are a number of limitations. The sequencing depth could be higher to enable the reconstruction of additional high-quality or near-complete MAGs, which will facilitate the identification of functional genes and putative BGCs. Although the genomes of a large number of known and novel species were retrieved, their ecological roles on the pier surfaces or in their indigenous environments could not be determined. The metabolic functions of the metagenomes have indicated the presence of functions such as xenobiotics biodegradation, but the surface chemical compositions and other properties were not determined, limiting our ability to understand the forces driving the colonization and metabolic activities of microbes on the different surfaces. The metagenomics results here have only revealed the metabolic potentials of the pier surface microbiomes, and future metatranscriptomics and/or metaproteomics analyses of samples over time will be required to investigate how the metabolically active microbial taxa adapt and survive the stresses under oligotrophic conditions.

## Conclusions

The inert and oligotrophic coastal outdoor pier surfaces contain diverse microorganisms from marine and anthropogenic sources, with surface material strongly affecting the taxonomic and functional compositions of the microbiomes. The taxa and metabolic functions in the microbiomes reflected some of the potential adaptation strategies and processes occurring on the surfaces such as corrosion and biodegradation of pollutants. The genomes of many novel bacterial species were reconstructed from the different surface types, surface materials, and geographical locations. Functional characterization of the MAGs highlighted the close association between taxonomy and their functional potential and biosynthetic capacity. Overall, this study has expanded our understanding of the taxonomy and the functional traits of microbial communities on outdoor surfaces of built environments.

## Materials and methods

### Sampling and metagenomic sequencing

One hundred and seventy-five samples were collected from surfaces of nine public piers located along the coastline in different parts of Hong Kong (Additional file 14: Fig. S9a) from June to July 2017. Dense vegetation is within close proximity to most piers. The average ambient temperature (30.7 ± 1.4 °C) and relative humidity (75.2 ± 6.7%) were measured for each location during sampling. Four types of surfaces including bollard, floor, handrail, and pole were sampled at each pier (Additional file 14: Fig. S9b) by swabbing an area of ~ 4 cm^2^ for 30 s using a Copan Liquid Amies Elution Swab (ESwab, Copan Diagnostics Inc., USA). The four surface types were further categorized according to surface material into either the concrete (i.e., bollard and floor) or the metal (i.e., handrail and pole) group. Based on visual inspection, the metal surfaces contained a mixture of iron and steel. All surfaces were dry, exposed to direct sunlight, and occasionally touched by users of the piers (except floor). Pitting corrosion was visible on all of the metal handrails and poles. All swabs were preserved in 1 mL of liquid Amies during transport and stored at − 80 °C upon arrival in the laboratory. Genomic DNA extraction and metagenomic sequencing were performed as described previously [3]. Twelve new swabs were processed in parallel with the surface samples as negative controls. An average of 9.6 million paired-end 125-bp raw reads were obtained per sample.

### Quality control and contaminant removal

Adapters were removed from the raw sequences using AdapterRemoval (v2.2.2) [75]. Quality filtering and trimming were performed using KneadData (https://bitbucket.org/biobakery/kneaddata/wiki/Home, v0.7.6) with default parameters and the human genome hg38 as the reference to remove human sequences. An average of 7.6 million paired-end reads per sample was retained for downstream steps. Co-assembly of reads from the 12 quality-filtered negative controls was performed using MetaWRAP (v1.2.1) [76] with MEGAHIT as the default assembly method and a minimum contig length of 1000 bp. Reads in the surface samples that could be mapped to the contigs in the negative controls were removed using an in-house script, and any unpaired reads were further removed from the paired-end fastq files using fastq-pair (https://github.com/linsalrob/fastq-pair). The algorithm decontam (https://github.com/benjjneb/decontam) executed using the default mode was further applied to evaluate contamination after the read removal procedures. The contaminating species identified were manually curated; however, it was not necessary to remove all of them because their relative abundance was low in the surface samples. After contaminants removal, an average of 6.4 million paired-end clean reads per sample was retained.

### Taxonomic classification and associations of species with metadata

The taxonomic profile of the surface metagenomes was annotated using Kraken2 [77] with the MiniKraken_v1_8GB database (April 2019 version). The relative abundance of species was further estimated using Bracken [78], and an average of 13.7% of reads could be classified to the species level. The associations between species abundance and parameters in the metadata including surface type, surface material, sampling location, ambient temperature and relative humidity, and sampling date were studied using MaAsLin2 (https://github.com/biobakery/Maaslin2). Only species with a mean relative abundance of ≥0.1% and a prevalence of ≥25% across all samples were included in the MaAsLin2 analysis.

### Identification of corrosion-related bacteria

A list of representative corrosion-related bacteria consisting of 22 genera and 34 species was compiled based on previous studies [31–34] (Additional file 2: Table S1). These bacteria can be categorized into five groups according to their corrosion mechanisms, which include iron-oxidizing bacteria (IOB), iron-reducing bacteria (IRB), nitrate-reducing bacteria associated with the redox cycling of iron (NRB-Fe), siderophore-producing NRB (NRB-S), and sulfate-reducing bacteria (SRB). The presence of corrosion-related bacteria in the pier samples was checked against the representative list.

### Alpha- and beta-diversity analyses

For the taxonomic alpha-diversity analysis, clean paired-end sequences were rarefied to 1.0 million reads per sample using the “seqtk” (v1.3-r106) [79] tool, reducing the dataset from 175 to 155 samples. Although the applied rarefaction depth was not sufficient to capture the richness of surface metagenomes for most samples (Additional file 15: Fig. S10), principal coordinate analysis indicated that the adopted depth could still recapitulate the compositional differences between microbial communities (Additional file 16: Fig. S11). At the species level, the abundance-based Shannon diversity index was calculated using the function “diversity” in the R (v3.6.1) package “vegan” (v2.5–6) [80]. The Bray–Curtis dissimilarity metric was calculated for the species-level taxonomic composition (unrarefied dataset) using the function “vegdist” in the R package “vegan.”

### Source tracking of surface metagenomes

The fast expectation-maximization microbial source tracking (FEAST) algorithm [81] was used to track the sources of microbial populations detected on the pier surfaces. To reflect local microbiome sources, 12 marine sediment samples collected near Hong Kong [82] and 20 skin samples of Chinese women [83] were used as the representative microbial sources. Other types of microbial sources were not considered owing to the lack of available local metagenomics data. Sequence processing, quality control, and taxonomic annotation of the source metagenomes were the same as the surface samples. FEAST was performed based on the species-level microbial taxa table using the R package “FEAST” (v1.0.1) with default parameters.

### Functional annotation of short reads

Gene families of the short reads in surface metagenomes were identified using HUMAnN2 [84] by checking against the UniRef90 database [85]. The species-level contributions to the functional shifts in the surface metagenomes were determined using FishTaco [86]. The analysis was performed on 146 species (average relative abundance of ≥0.1% across samples) and 2093 KOs (average copy per million of ≥100). The reference genomes of the 146 species were downloaded from the NCBI microbial genomes resource, and the open reading frame of the genomes was converted into protein sequences using prodigal (v2.6.3) [87]. Each of the 2093 KOs was assigned to the protein sequences using KofamKOALA (v1.3.0) [88]. FishTaco was performed using the genomic content inference mode and the “single_taxa” assessment method.

### Assembly of contigs and reconstruction of MAGs

The reads of each sample were assembled into contigs by MetaWRAP (v1.2.1) [76] with megahit as the assembly method and a minimum contig length of 1000 bp. The resulting contigs were binned into MAGs using the MetaWRAP “binning” module with three different binning algorithms (i.e., metabat2, maxbin2, and concoct). The resulting MAGs were further refined using the MetaWRAP “bin_refinement” module. After refinement, 150 MAGs with various levels of completeness (75 to 100%) and contamination (0 to 5%) were obtained. Dereplication was not performed for the 150 MAGs because we aimed to understand the distribution pattern and functional traits of all the MAGs across different surface types and the highly similar MAGs may harbor different sets of BGCs (this difference will be lost if dereplication is performed). The read coverage of the MAGs was calculated by CoverM (v0.4.0), which ranged between 1.1 and 25% per sample, with an average of 5.7%. The taxonomy of the 150 MAGs was annotated using GTDB-TK (v1.3.0) [89] (Additional file 17: Table S6). The ANI values between the MAGs and their closest reference genomes were calculated using fastANI [90]. The phylogeny of the MAGs was studied using PhyloPhlAn3 [91] and visualized using the Interactive Tree of Life tool (ITOL, https://itol.embl.de).

### Functional annotation of contigs and MAGs

The open reading frames of the contigs in each sample and the MAGs were predicted using Prokka (v1.14.6) [92] and the functions of the translated protein sequences were annotated using EggNOG-mapper (v2.0.1) [93]. The Jaccard distance index was applied to study the compositional differences between samples based on the presence/absence of functional genes or the KOs encoding xenobiotic biodegradation and metabolism pathways using the function “vegdist” in the R package “vegan.” The resulting pairwise distance matrix was subjected to two-way hierarchical clustering analysis with the function “heatmap” in the R package “heatmap3” (v1.1.7). The supervised random forest algorithm was implemented using the R package “randomForest” [94] (v4.6.14) to assess the performance of the hierarchical clustering in classifying the samples, with the classification accuracy evaluated by the out-of-bag error. The metabolic functions in the contigs that are related to microbial iron utilization were annotated using FeGenie [34]. The biosynthesis genetic clusters in the MAGs were predicted using antiSMASH (v4.2.0) [95].

### Statistical analysis

The Mann–Whitney and Kruskal–Wallis tests were performed to test the statistical significance involving two and more than two groups using the “wilcox.test” and “kruskal.test” functions of the R package “stats” (v3.6.1), respectively. The post hoc Kruskal–Wallis test was performed using the “kruskalmc” function of the R package “pgirmess” (v1.6.9) [96]. Because only a single MAG was reconstructed from the phyla Acidobacteria, Firmicutes, and Gemmatimonadetes, they were excluded from the Kruskal–Wallis test when testing the statistical significance of the antiSMASH results. The Procrustes test was preformed using the “protest” function in the R package “vegan” with 999 permutations.

A stepwise model selection scheme based on the Shannon diversity index was applied to identify factors that significantly affected the within-sample diversity of surface microbiomes. In the analysis, each parameter in the metadata and all possible two-way interactions of these parameters were set as predictors of the Shannon diversity index score in the linear mode using the “lm” function in the R package “stats.” A stepwise model selection was applied with the “stepAIC” function in the R package “MASS” (v7.5–51.5) [97] and the model with the lowest Akaike information criterion (AIC) value was considered to be optimal. The significance of each parameter in the optimal linear model was calculated using the “anova” function in the R package “stats.”

The Bray–Curtis dissimilarity of species-level compositional differences between samples was analyzed by applying the permutational multivariate analysis of variance (PERMANOVA) test using the “adonis2” function in the R package “vegan.” All of the parameters in the metadata and all two-way interactions of the parameters were set as predictors of the Bray–Curtis dissimilarity matrix in the linear model for PERMANOVA. The AIC value of the model was calculated by manually removing one variable at a time until the next removal resulted in no increase in the AIC value.

## Supplementary Information

**Additional file 1: Figure S1**. Differentially enriched species between the concrete and metal surface microbiomes. Seventeen species were identified by the algorithm MaAsLin2 to be differentially associated with surface materials. The statistical significance was corrected using the Benjamini–Hochberg method.

**Additional file 2: Table S1**. List of representative corrosion-related bacteria.

**Additional file 3: Table S2**. Statistics of alpha-diversity analysis.

**Additional file 4: Table S3**. Statistics of beta-diversity analysis.

**Additional file 5: Figure S2**. Species that contributed to pathways related to xenobiotics biodegradation and metabolism as well as energy metabolism on pier surfaces.

**Additional file 6: Figure S3**. Relative abundance of COG categories between the metal and concrete microbiomes. Each point represents a sample, colored by surface type. All pairwise comparisons were statistically significant (MW test, *p* < 0.05) except for categories [G], [I], and [T].

**Additional file 7: Figure S4**. Relative abundance of COG categories in the gene cluster D. The functional genes highly conserved in gene cluster D were grouped according to the COG categories.

**Additional file 8: Figure S5**. Xenobiotic metabolic functions of the pier surface microbiomes differed by surface material. Two-way hierarchical clustering of KOs associated with xenobiotic metabolism identified in the contigs of each sample. KOs that are present or absent are indicated by the dark and light blue colors, respectively. KOs (column) were hierarchically clustered according to their presence/absence in the samples. The four clusters of the vertical dendrogram are highlighted.

**Additional file 9: Figure S6**. Relative abundance and composition of iron-related functions in the pier surface microbiomes. (a) Relative abundance of four categories of iron-related genes delineated by surface type and material. Statistics cannot be determined for genes associated with iron oxidation owing to insufficient samples for the two surface materials. (b) Principal coordinate analysis of the Bray–Curtis dissimilarity based on the abundance and membership of iron-related proteins in the surface microbiomes.

**Additional file 10: Figure S7**. Genomic comparison between each MAG and its closest relative. Each point indicates the average nucleotide identity (ANI) value (x-axis) and the alignment fraction (y-axis) between an MAG and its closest relative according to the GTDB database. The MAGs without the closest genomic relative were excluded. The ANI threshold used for species delineation is 95% (blue dotted line). MAGs that were below the threshold could not be assigned to a known species.

**Additional file 11: Table S4**. Influence of taxonomy, surface type, and geography on the relative abundance of COG categories.

**Additional file 12: Figure S8**. Relative abundance of COG categories in the MAGs differed by phylum. The distribution of the data and its probability density are indicated by a violin plot, and the interquartile range of the data is shown by a standard boxplot. Differences between phyla were statistically significant (KW test, *p* < 0.05) for all COG categories except [Z]. The statistical *p*-value for each individual COG comparison is provided in Table S4. For Acidobacteria, Firmicutes, and Gemmatimonadetes (each with a single MAG), the relative abundance in each COG category is indicated by a gray point. The absence of any symbol represents a COG category that is not found in a phylum.

**Additional file 13: Table S5**. Influence of taxonomy, surface type, and geography on the relative abundance of the types of putative BGCs.

**Additional file 14: Figure S9**. A map of the sampling locations and photos of the surface types. (a) Location of the nine piers and (b) representative photos of the four surface types.

**Additional file 15: Figure S10**. Rarefaction curves of the pier surface metagenomes. Each line represents the number of species identified in a sample at a given sequencing depth. The adopted rarefaction depth is indicated by the vertical line.

**Additional file 16: Figure S11**. Influence of rarefaction on the surface microbiome compositional differences. Principal coordinate analysis of the rarefied (left) and unrarefied (right) surface microbiomes based on the species-level abundance matrix ordinated by the Bray–Curtis dissimilarity metric. The normal confidence ellipses indicate the confidence level at 95%.

**Additional file 17: Table S6**. Taxonomic classification of the MAGs according to the GTDB database.

## Data Availability

Sequencing reads generated for this project have been deposited in NCBI under BioProject accession number PRJNA722771. Computational scripts that were used in this study are publicly available in FigShare (10.6084/m9.figshare.14413052).
