# Supplementary material for: M﻿etagenomic insights into the microbial communities of inert and oligotrophic outdoor pier surfaces of a coastal city
Source: Microbiome. 2021 Nov 2;9:213. doi: 10.1186/s40168-021-01166-y (PMC8562002; doi:10.1186/s40168-021-01166-y)
Supplement: Supplementary file 4 — Additional file 3: Table S2. Statistics of alpha-diversity analysis. [file 40168_2021_1166_MOESM3_ESM.docx]

**Additional file 3: Table S2.** **Statistics of alpha-diversity analysis.**

| Predictor | DF | Sum Sq. | Mean Sq. | pseudo-*F* | R^2^ | *p*-value |
| --- | --- | --- | --- | --- | --- | --- |
| Surface type | 3 | 12.82 | 4.27 | 20.27 | 0.16 | 1.00 × 10^-10^ |
| Location | 8 | 5.79 | 0.72 | 3.43 | 0.07 | 0.001 |
| Surface type: Location | 21 | 38.27 | 1.82 | 8.65 | 0.46 | 1.04 × 10^-15^ |
| Residuals | 122 | 25.71 | 0.21 |  | 0.31 |  |

The best-fit model for Shannon’s diversity index score. The model explained 69% of within-sample diversity variance. Only statistically significant predictors are shown.
