# Supplementary material for: M﻿etagenomic insights into the microbial communities of inert and oligotrophic outdoor pier surfaces of a coastal city
Source: Microbiome. 2021 Nov 2;9:213. doi: 10.1186/s40168-021-01166-y (PMC8562002; doi:10.1186/s40168-021-01166-y)
Supplement: Supplementary file 5 — Additional file 4: Table S3. Statistics of beta-diversity analysis. [file 40168_2021_1166_MOESM4_ESM.docx]

**Additional file 4: Table S3.** **Statistics of beta-diversity analysis.**

| Predictor | DF | Sum Sq. | pseudo-*F* | R^2^ | *p*-value |
| --- | --- | --- | --- | --- | --- |
| Surface material | 1 | 6.14 | 117.61 | 0.17 | 0.001 |
| Surface type | 2 | 2.25 | 21.55 | 0.06 | 0.001 |
| Location | 8 | 7.60 | 18.21 | 0.21 | 0.001 |
| Surface type: Location | 24 | 12.51 | 9.99 | 0.35 | 0.001 |
| Residuals | 138 | 7.20 |  | 0.20 |  |

The best-fit PERMANOVA model for composition differences between the surface microbiomes. The model explained 80% of between sample composition differences based on the Bray-Curtis dissimilarity. Only statistically significant predictors are shown.
