# Supplementary figures and images for: M﻿etagenomic insights into the microbial communities of inert and oligotrophic outdoor pier surfaces of a coastal city
Source: Microbiome. 2021 Nov 2;9:213. doi: 10.1186/s40168-021-01166-y (PMC8562002; doi:10.1186/s40168-021-01166-y)

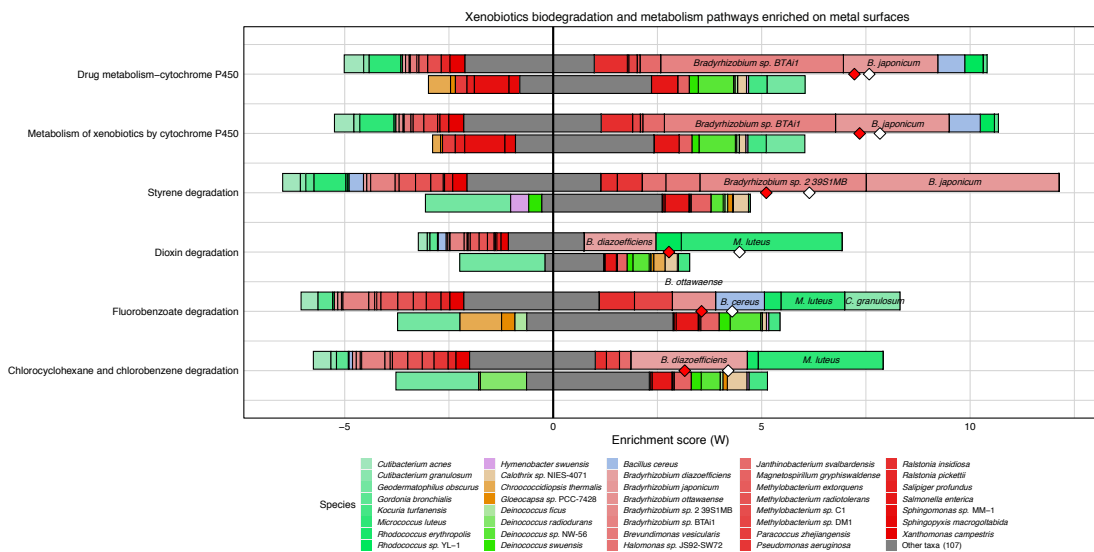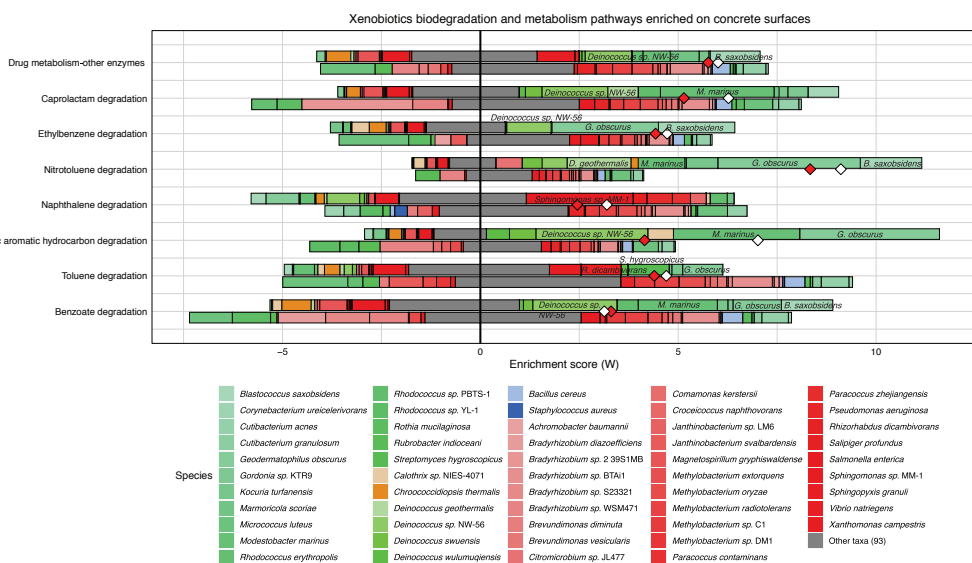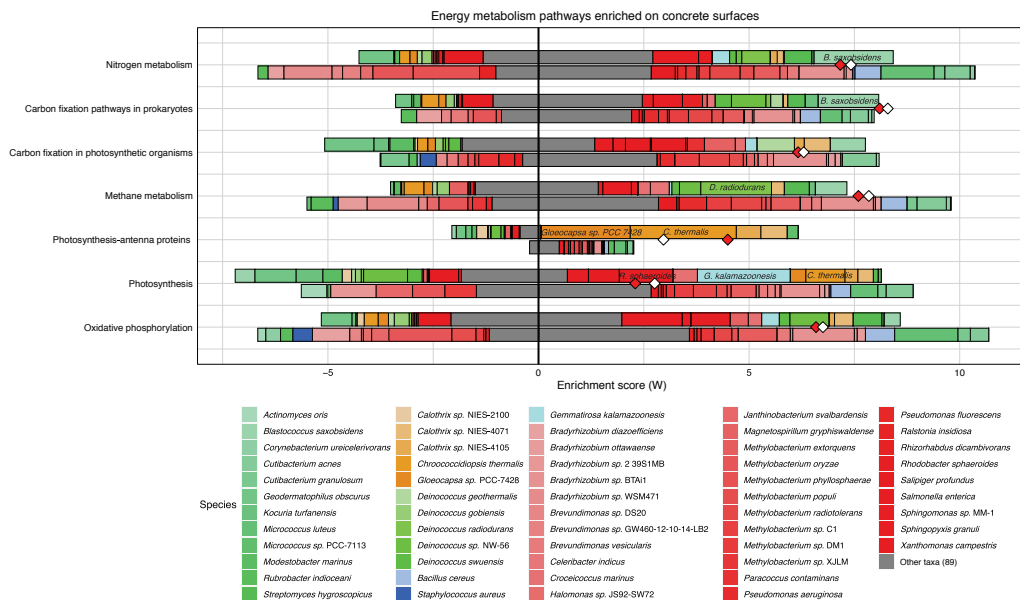

Supplement: Supplementary file 6 — Additional file 5: Figure S2. Species that contributed to pathways related to xenobiotics biodegradation and metabolism as well as energy metabolism on pier surfaces. [file 40168_2021_1166_MOESM5_ESM.pdf]

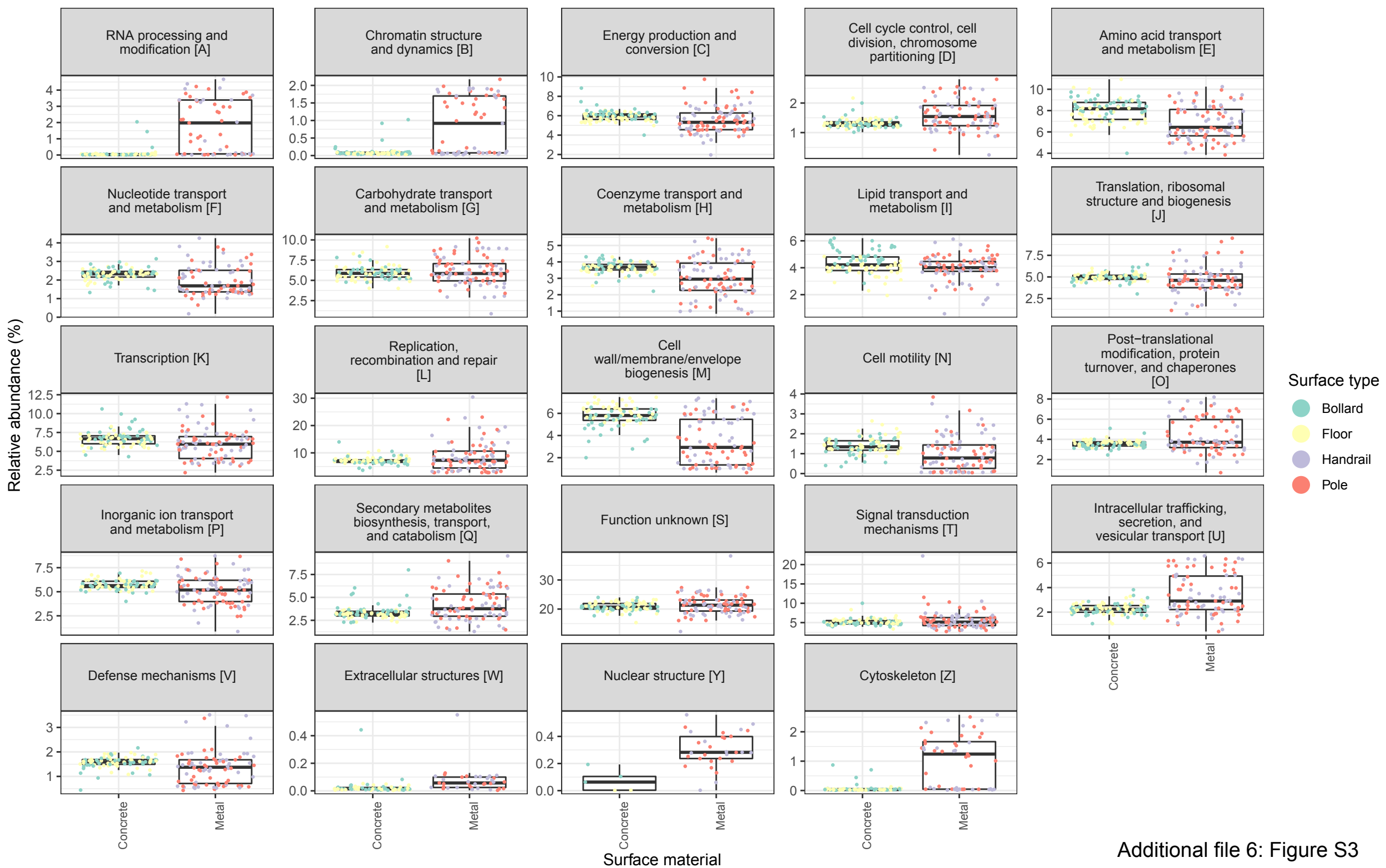

Supplement: Supplementary file 7 — Additional file 6: Figure S3. Relative abundance of COG categories between the metal and concrete microbiomes. Each point represents a sample, colored by surface type. All pairwise comparisons were statistically significant (MW test, p < 0.05) except for categories [G], [I], and [T]. [file 40168_2021_1166_MOESM6_ESM.pdf]

Relative abundance (%)

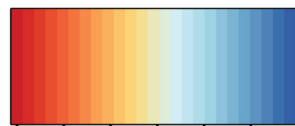

0 2 4 6 8 10

Surface type

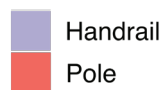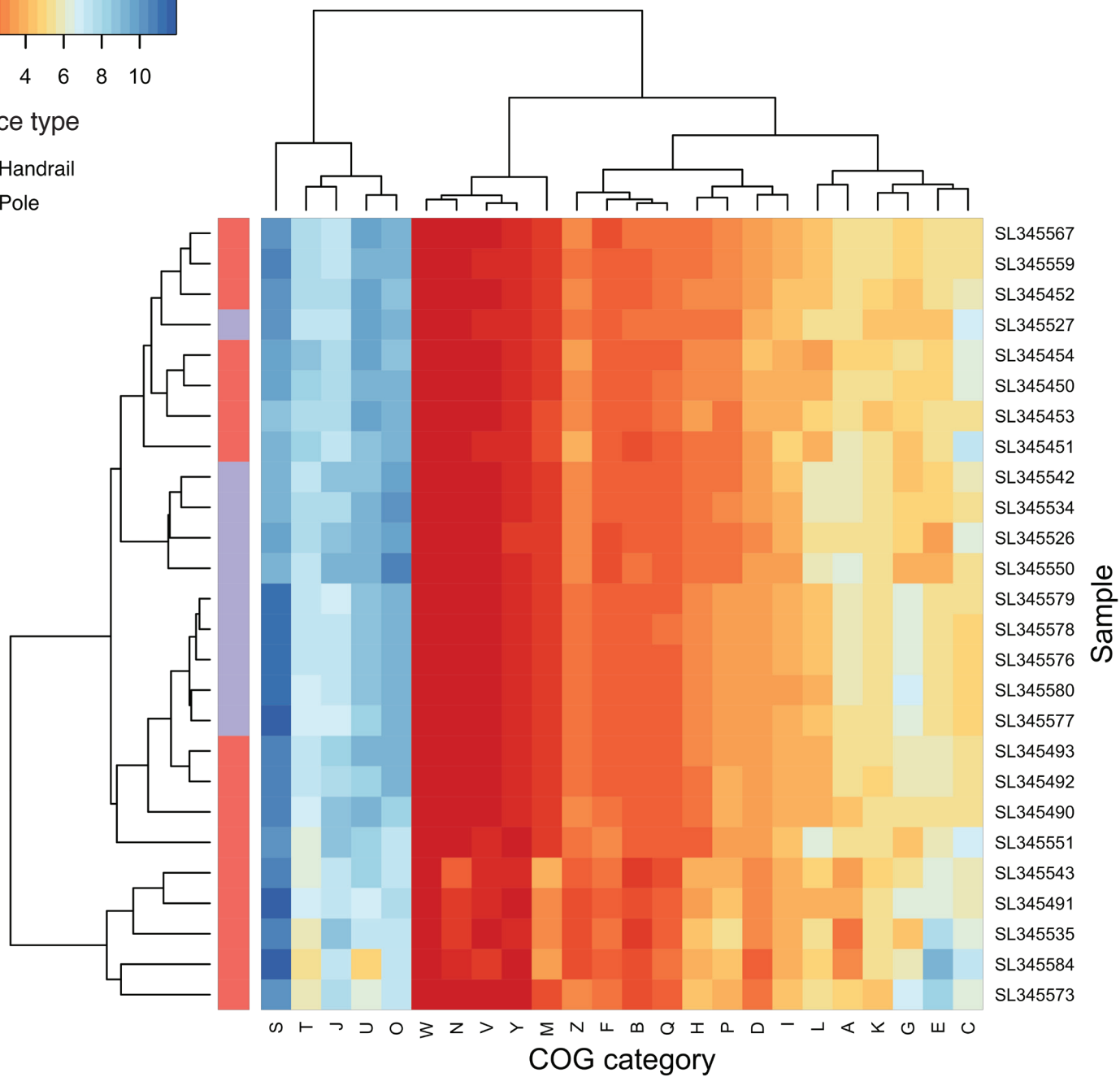

Supplement: Supplementary file 8 — Additional file 7: Figure S4. Relative abundance of COG categories in the gene cluster D. The functional genes highly conserved in gene cluster D were grouped according to the COG categories. [file 40168_2021_1166_MOESM7_ESM.pdf]

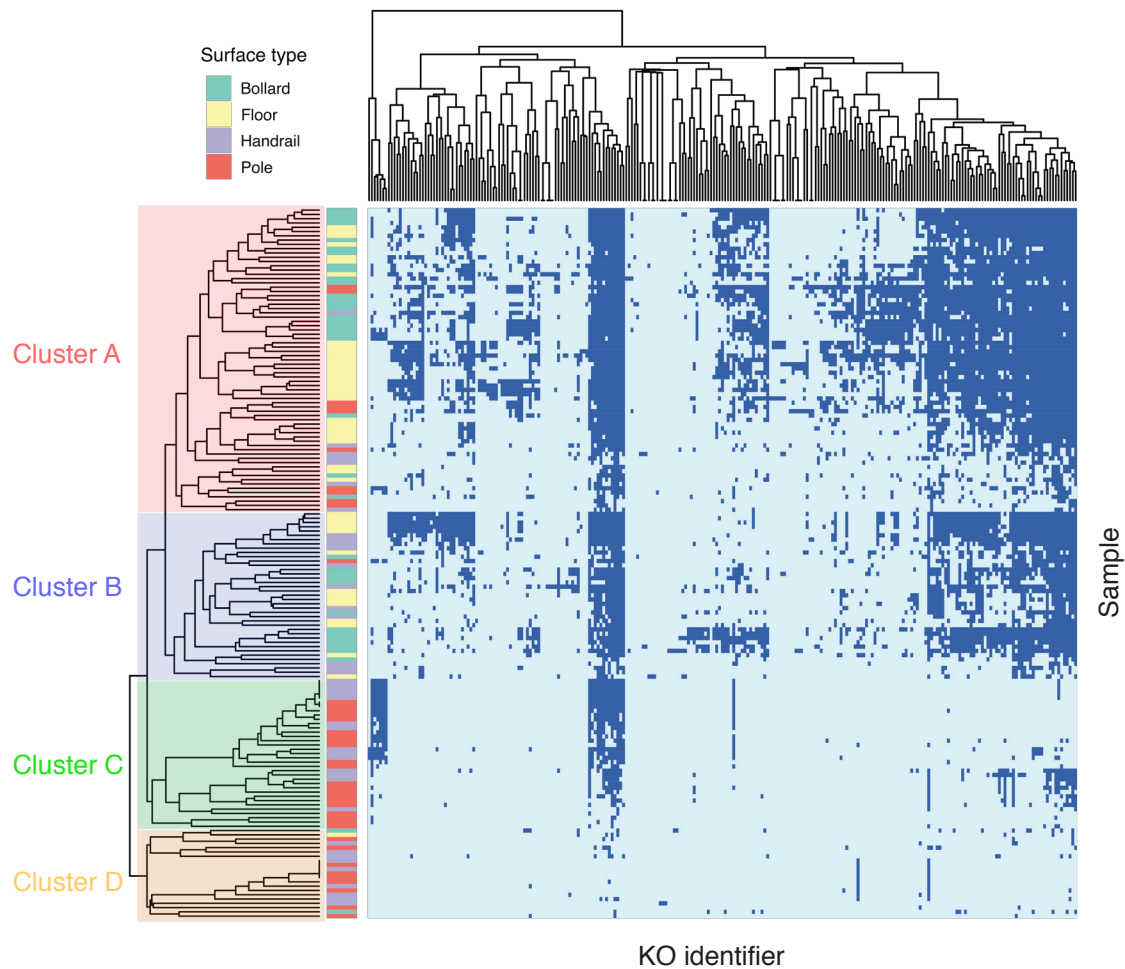

Supplement: Supplementary file 9 — Additional file 8: Figure S5. Xenobiotic metabolic functions of the pier surface microbiomes differed by surface material. Two-way hierarchical clustering of KOs associated with xenobiotic metabolism identified in the contigs of each sample. KOs that are present or absent are indicated by the dark and light blue colors, respectively. KOs (column) were hierarchically clustered according to their presence/absence in the samples. The four clusters of the vertical dendrogram are highlighted. [file 40168_2021_1166_MOESM8_ESM.pdf]

a

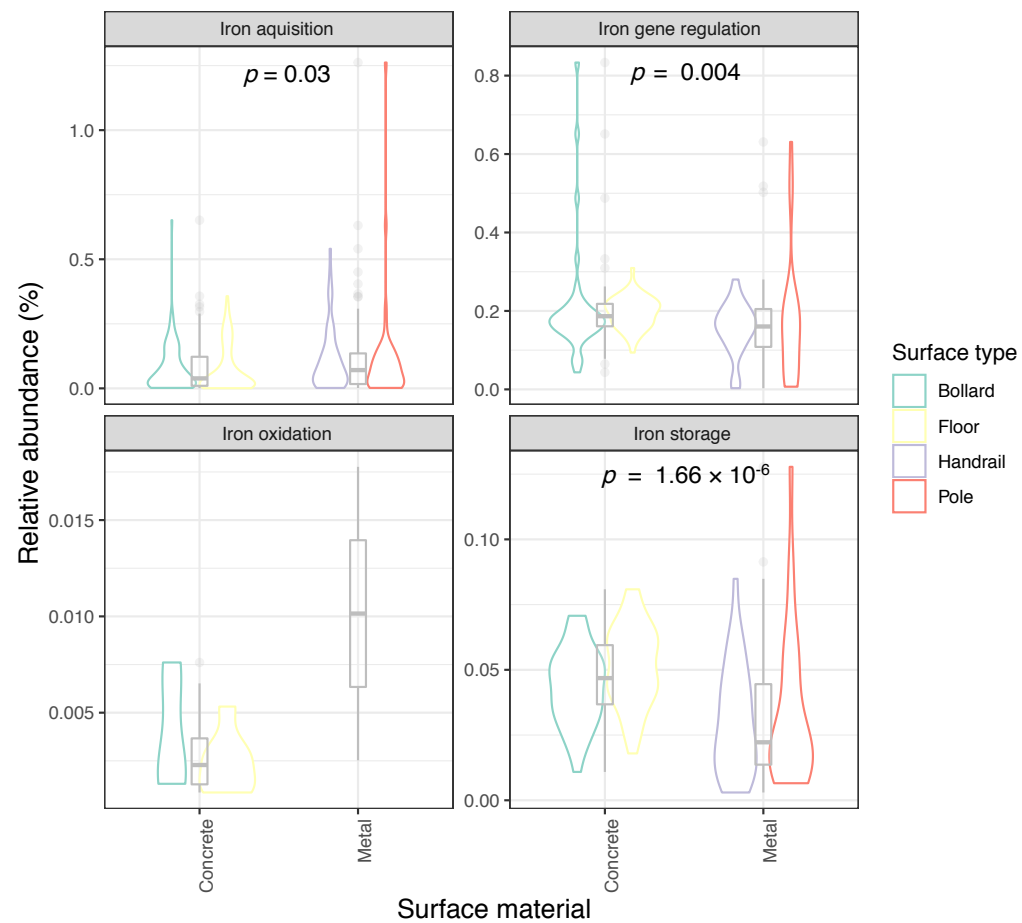

b

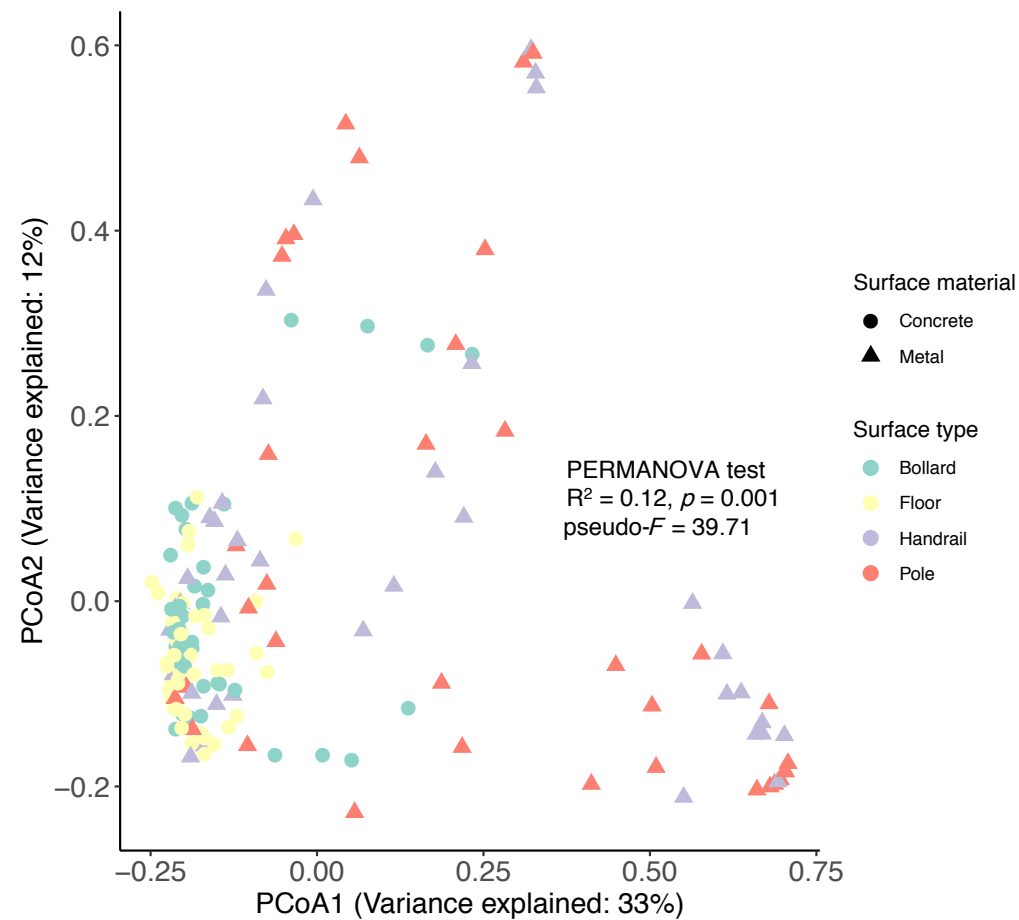

Supplement: Supplementary file 10 — Additional file 9: Figure S6. Relative abundance and composition of iron-related functions in the pier surface microbiomes. (a) Relative abundance of four categories of iron-related genes delineated by surface type and material. Statistics cannot be determined for genes associated with iron oxidation owing to insufficient samples for the two surface materials. (b) Principal coordinate analysis of the Bray–Curtis dissimilarity based on the abundance and membership of iron-related proteins in the surface microbiomes. [file 40168_2021_1166_MOESM9_ESM.pdf]

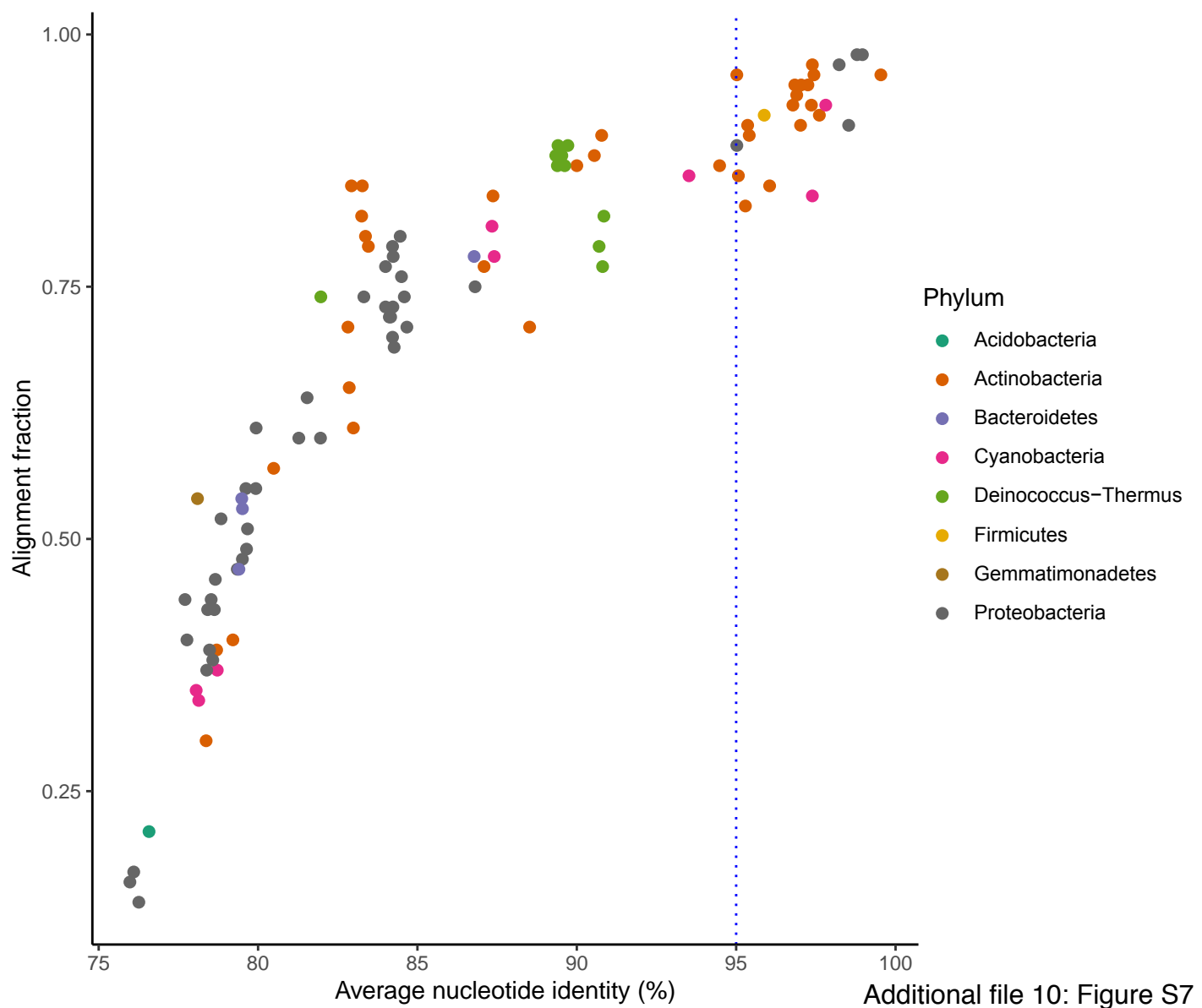

Supplement: Supplementary file 11 — Additional file 10: Figure S7. Genomic comparison between each MAG and its closest relative. Each point indicates the average nucleotide identity (ANI) value (x-axis) and the alignment fraction (y-axis) between an MAG and its closest relative according to the GTDB database. The MAGs without the closest genomic relative were excluded. The ANI threshold used for species delineation is 95% (blue dotted line). MAGs that were below the threshold could not be assigned to a known species. [file 40168_2021_1166_MOESM10_ESM.pdf]

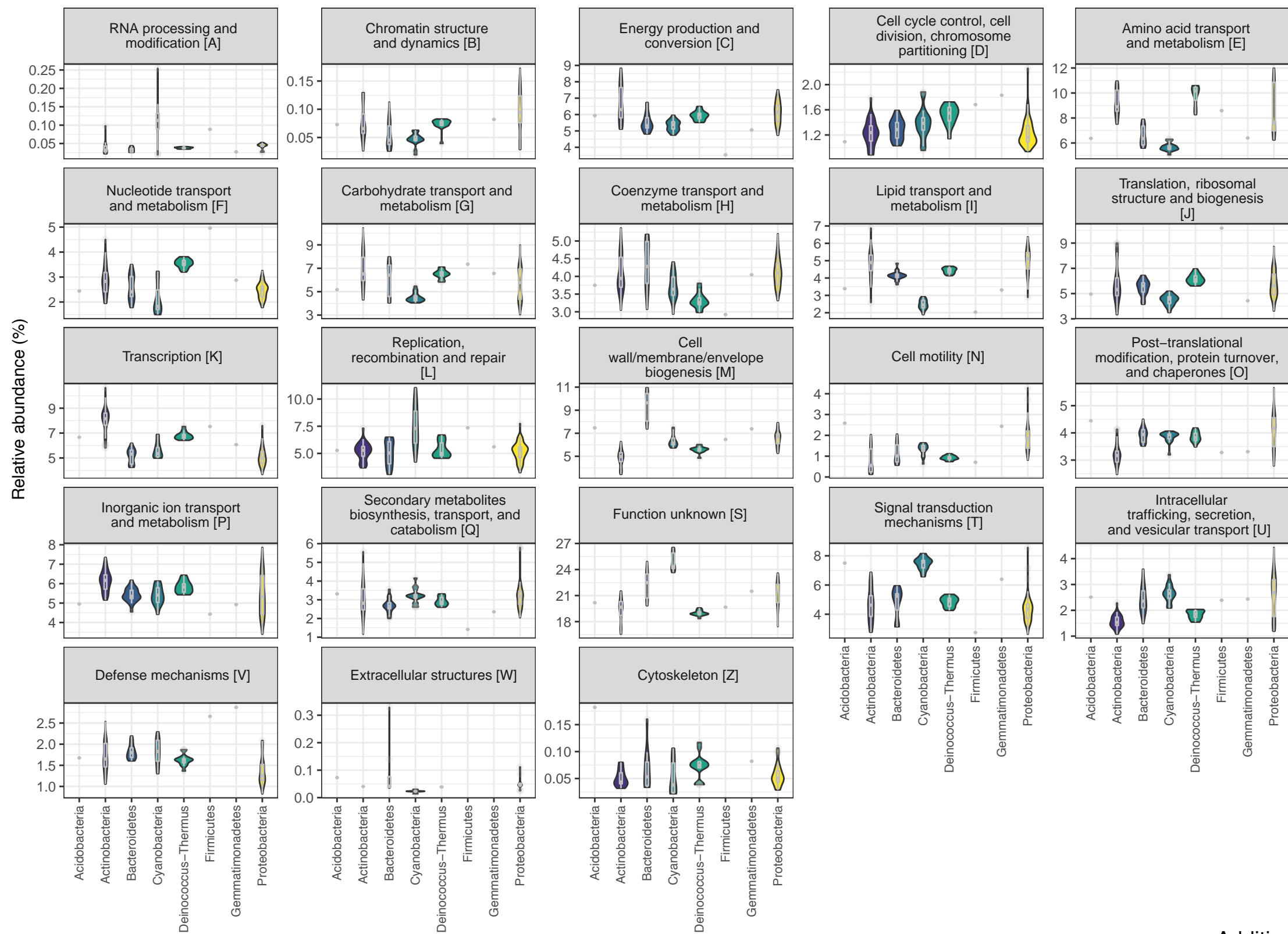

Supplement: Supplementary file 13 — Additional file 12: Figure S8. Relative abundance of COG categories in the MAGs differed by phylum. The distribution of the data and its probability density are indicated by a violin plot, and the interquartile range of the data is shown by a standard boxplot. Differences between phyla were statistically significant (KW test, p < 0.05) for all COG categories except [Z]. The statistical p-value for each individual COG comparison is provided in Table S4. For Acidobacteria, Firmicutes, and Gemmatimonadetes (each with a single MAG), the relative abundance in each COG category is indicated by a gray point. The absence of any symbol represents a COG category that is not found in a phylum. [file 40168_2021_1166_MOESM12_ESM.pdf]

a

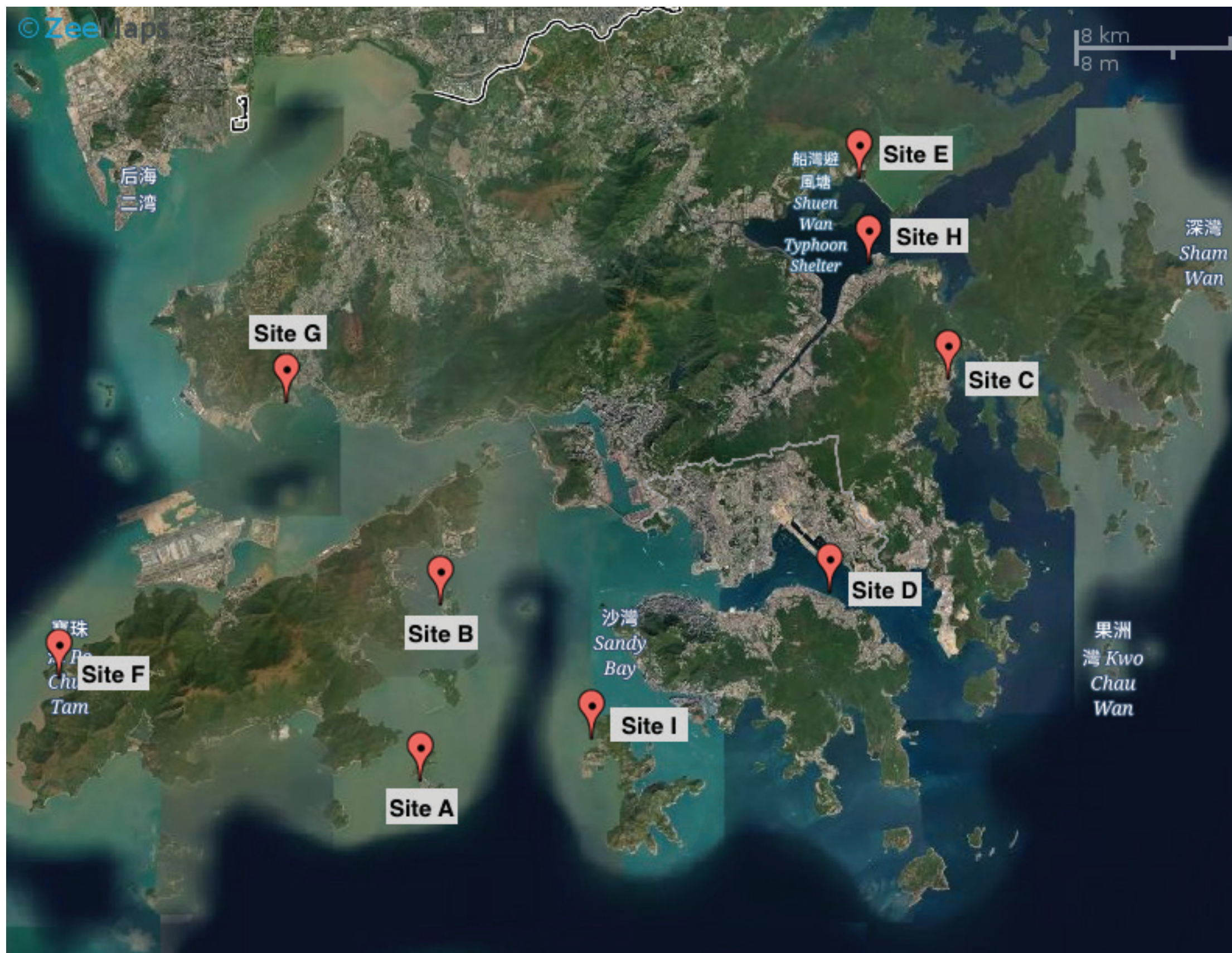

b

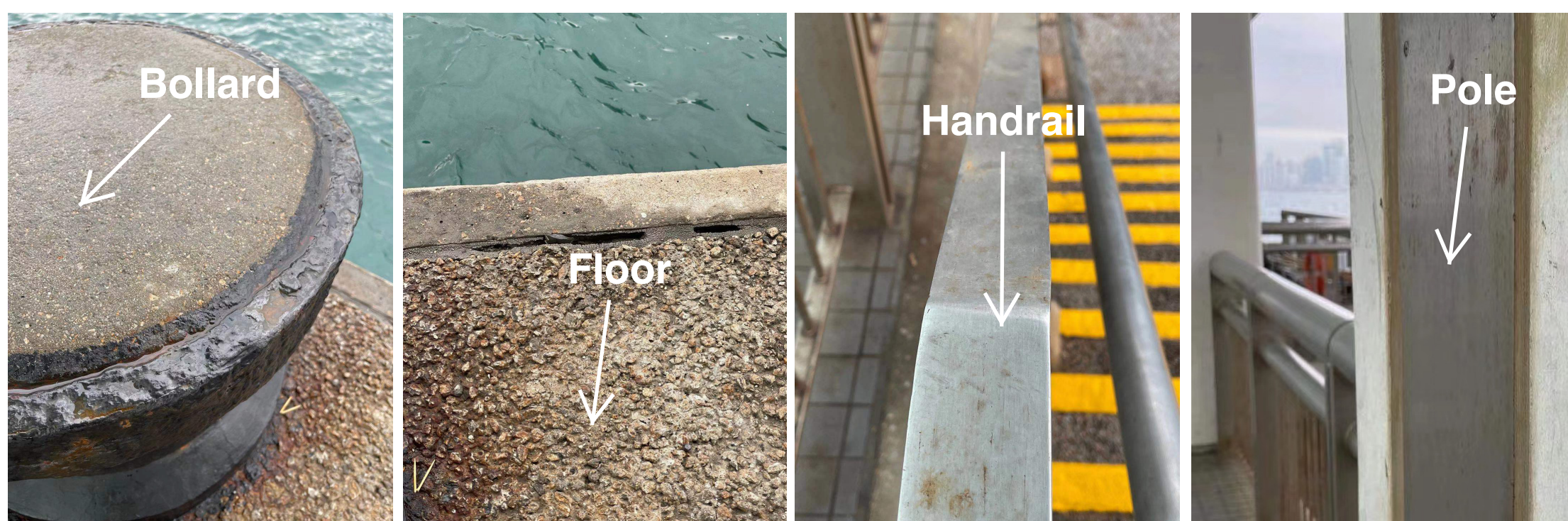

Supplement: Supplementary file 15 — Additional file 14: Figure S9. A map of the sampling locations and photos of the surface types. (a) Location of the nine piers and (b) representative photos of the four surface types. [file 40168_2021_1166_MOESM14_ESM.pdf]

Rarefied depth = 1,017,263

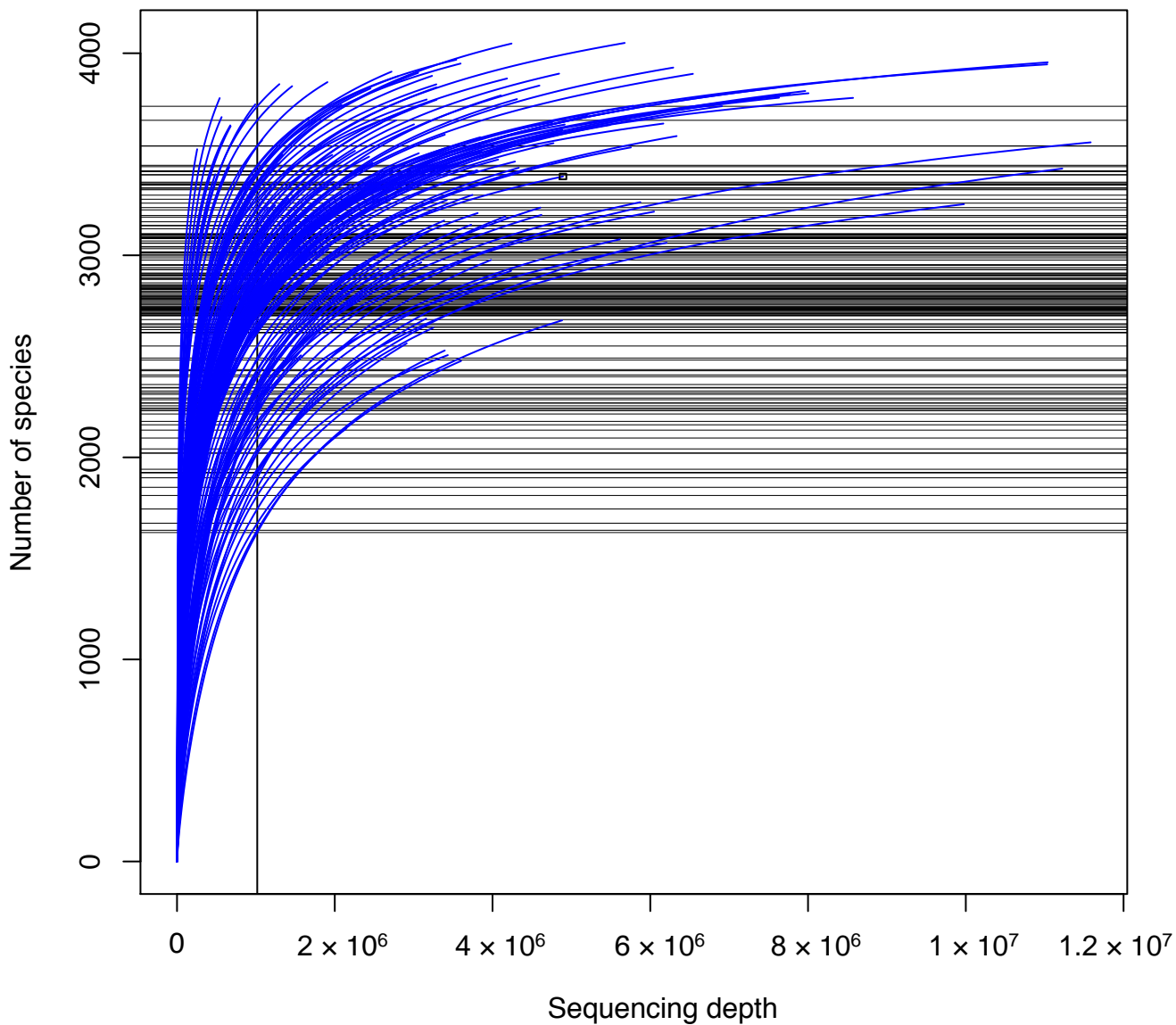

Supplement: Supplementary file 16 — Additional file 15: Figure S10. Rarefaction curves of the pier surface metagenomes. Each line represents the number of species identified in a sample at a given sequencing depth. The adopted rarefaction depth is indicated by the vertical line. [file 40168_2021_1166_MOESM15_ESM.pdf]

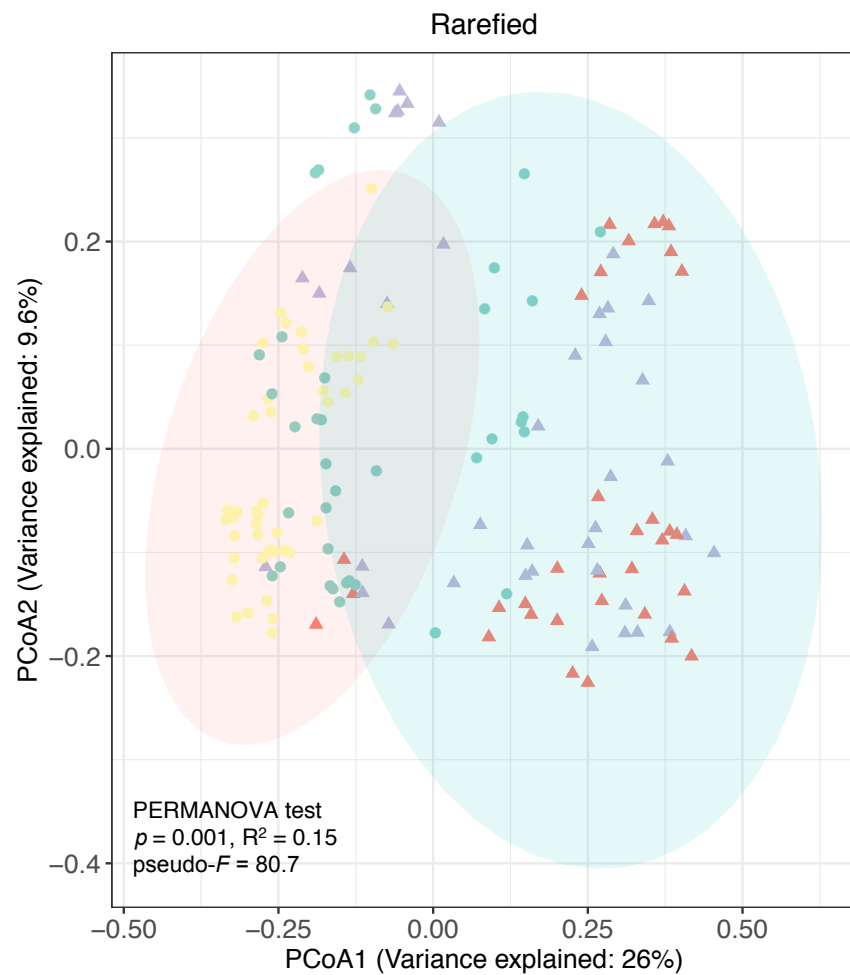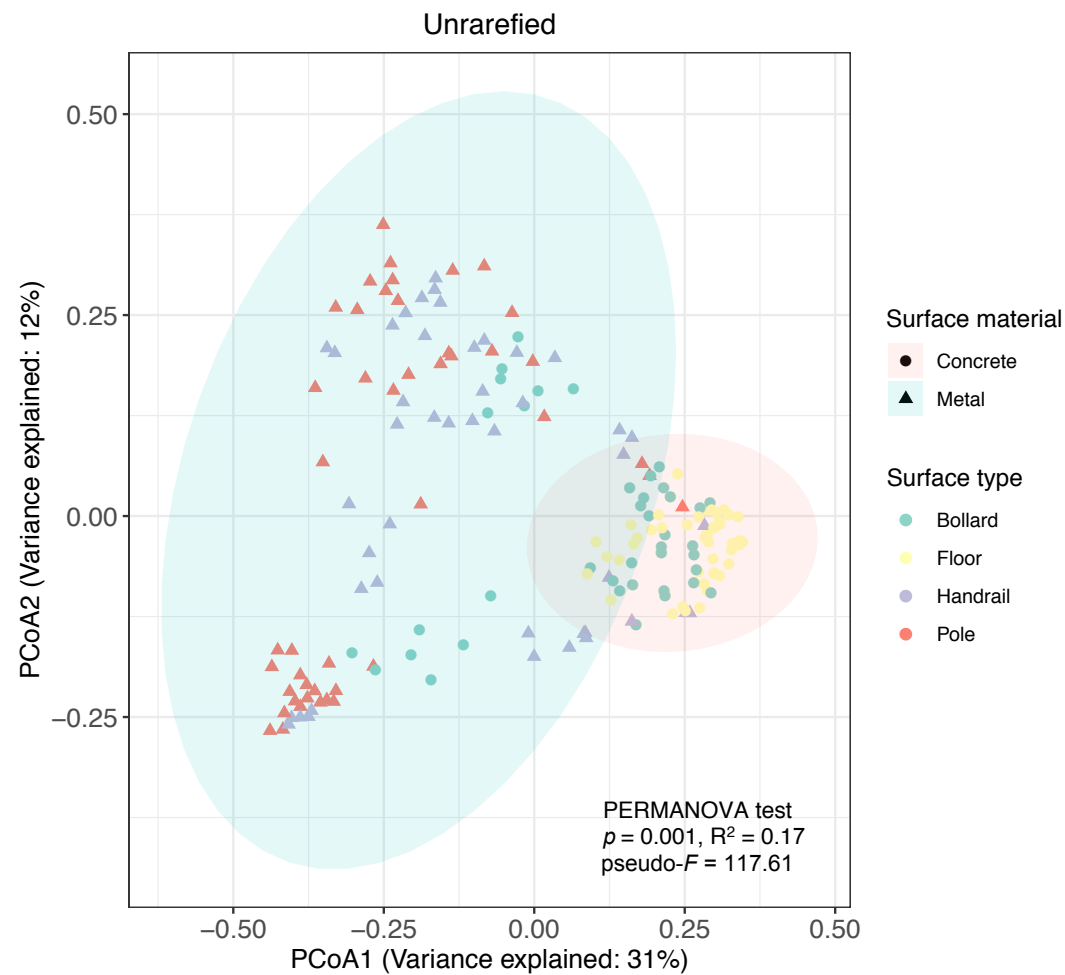

Additional file 16: Figure S11

Supplement: Supplementary file 17 — Additional file 16: Figure S11. Influence of rarefaction on the surface microbiome compositional differences. Principal coordinate analysis of the rarefied (left) and unrarefied (right) surface microbiomes based on the species-level abundance matrix ordinated by the Bray–Curtis dissimilarity metric. The normal confidence ellipses indicate the confidence level at 95%. [file 40168_2021_1166_MOESM16_ESM.pdf]
