# Supplementary material for: M﻿etagenomic insights into the microbial communities of inert and oligotrophic outdoor pier surfaces of a coastal city
Source: Microbiome. 2021 Nov 2;9:213. doi: 10.1186/s40168-021-01166-y (PMC8562002; doi:10.1186/s40168-021-01166-y)
Supplement: Supplementary file 2 — Additional file 1: Figure S1. Differentially enriched species between the concrete and metal surface microbiomes. Seventeen species were identified by the algorithm MaAsLin2 to be differentially associated with surface materials. The statistical significance was corrected using the Benjamini–Hochberg method. [file 40168_2021_1166_MOESM1_ESM.pdf]

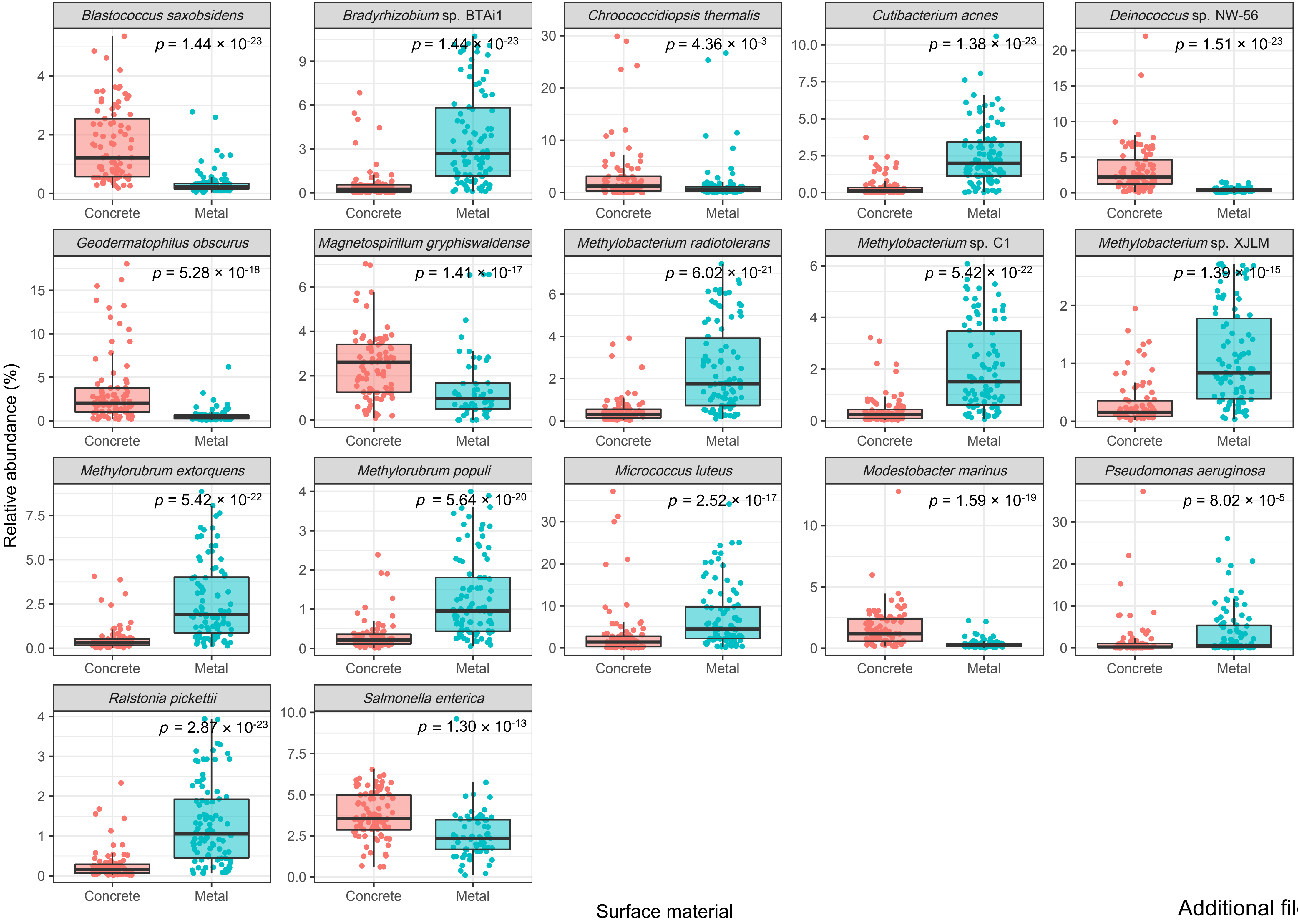

Additional file 1: Figure S1
